# Complete Chloroplast Genome of the Multifunctional Crop Globe Artichoke and Comparison with Other Asteraceae

**DOI:** 10.1371/journal.pone.0120589

**Published:** 2015-03-16

**Authors:** Pasquale L. Curci, Domenico De Paola, Donatella Danzi, Giovanni G. Vendramin, Gabriella Sonnante

**Affiliations:** 1 Institute of Biosciences and Bioresources, National Research Council, Bari, Italy; 2 Institute of Biosciences and Bioresources, National Research Council, Sesto Fiorentino (FI), Italy; Illinois Institute of Technology, UNITED STATES

## Abstract

With over 20,000 species, Asteraceae is the second largest plant family. High-throughput sequencing of nuclear and chloroplast genomes has allowed for a better understanding of the evolutionary relationships within large plant families. Here, the globe artichoke chloroplast (cp) genome was obtained by a combination of whole-genome and BAC clone high-throughput sequencing. The artichoke cp genome is 152,529 bp in length, consisting of two single-copy regions separated by a pair of inverted repeats (IRs) of 25,155 bp, representing the longest IRs found in the Asteraceae family so far. The large (LSC) and the small (SSC) single-copy regions span 83,578 bp and 18,641 bp, respectively. The artichoke cp sequence was compared to the other eight Asteraceae complete cp genomes available, revealing an IR expansion at the SSC/IR boundary. This expansion consists of 17 bp of the *ndhF* gene generating an overlap between the *ndhF* and *ycf1* genes. A total of 127 cp simple sequence repeats (cpSSRs) were identified in the artichoke cp genome, potentially suitable for future population studies in the *Cynara* genus. Parsimony-informative regions were evaluated and allowed to place a *Cynara* species within the Asteraceae family tree. The eight most informative coding regions were also considered and tested for “specific barcode” purpose in the Asteraceae family. Our results highlight the usefulness of cp genome sequencing in exploring plant genome diversity and retrieving reliable molecular resources for phylogenetic and evolutionary studies, as well as for specific barcodes in plants.

## Introduction

*Cynara cardunculus* L. is a complex species belonging to the second largest family of plants, Asteraceae, with over 20,000 species [1]. It includes two crops, the globe artichoke [*C*. *cardunculus* L. var. *scolymus* (L.) Fiori] and the cultivated or leafy cardoon (*C*. *cardunculus* var. *altilis* DC), and the wild cardoon (*C*. *cardunculus* var. *sylvestris* Lam.). The wild perennial cardoon has been recognized as the ancestor of both cultigens [2,3].

The globe artichoke is a diploid outcrossing crop (2n = 2x = 34) originating in the Mediterranean region. It fulfills an important role in human nutrition in this area, where it is mainly consumed as a vegetable for its large and edible immature flower heads. The globe artichoke is also well known for its beneficial properties, due to a high content in polyphenols such as flavonoids, caffeic acid, chlorogenic acid and cynarin [4,5,6], and to a particular abundance of inulin in roots [7]. Due to the high level of heterozygosity in its genome, for centuries the artichoke has been mainly asexually propagated in order to ensure commercial uniformity [8]. Recently, an increasing number of seed-propagated varieties have been released [9]. Artichoke cultivation is mainly located in Europe (where Italy is the leading producer) and in North Africa. More recently it has spread in California, Peru and Argentina (http://faostat.fao.org, 2012).

Despite much interest in the phylogenetics of Asteraceae, several relationships still need to be clarified. The Cynareae, Dicomeae, and Tarchonantheae formed a well-supported trichotomy in the cp metatree by Panero and Funk [10], which did not include any *Cynara* type species, but the relationships within the Cynareae (synonymous Cardueae) and between the Cynareae and the other tribes of the Carduoideae subfamily are not as well resolved [10,11].

Chloroplasts, which originate from ancient eubacterial invasions [12], are multifunctional organelles possessing their own genetic material. In most higher plants, including Angiosperms, the chloroplast (cp) genome forms a double stranded, circular molecule ranging from 120 to 160 kb that is highly conserved in size, structure and gene content [13,14]. The plant cp genomes typically harbor a quadripartite structure consisting of two inverted repeats (IRs) separated by two regions of unique DNA, the large (LSC) and small (SSC) single-copy regions [15]. Substitution rates in plant cp genomes are much lower than those in their nuclear genomes [16] and the very low level of recombination and primarily uniparental inheritance makes cp genomes a valuable source of genetic markers for phylogenetic analyses [17,18]. For these reasons, cp genomes are also useful tools for DNA barcoding. While universal DNA barcodes are not available for plants [19], Li *et al*. [20] recently proposed the use of taxon-specific barcodes for species identification using dedicated DNA cp-regions that have a sufficiently high mutation rate.

Since the publication of the first cp genome [21], the number of complete cp genomes available (http://www.ncbi.nlm.nih.gov/genome/) has increased rapidly thanks to the development of high-throughput technologies [20]. However, only a few representatives from the Asteraceae family have been completely sequenced and analyzed. Here we present the complete cp genome sequence of the globe artichoke, obtained by a combination of data retrieved from genome and BAC clone sequencing. This is the first published cp genome belonging to the subfamily Carduoideae and thus represents a solid resource for phylogenetic studies and comparative genomics of the Asteraceae. In this manuscript, we also searched for the most valuable regions for barcoding with potential applications across the large family of Asteraceae.

## Materials and Methods

### Chloroplast sequencing and analyses

Genomic DNA was extracted from young leaves of globe artichoke, variety “Brindisino” according to Sonnante *et al*. [22]. Whole genomic DNA was sent to IGA Technology Services (Udine, Italy) in order to perform Illumina sequencing, using the GAIIx platform (200–350 bp library insert size, 75 bp paired-end reads). Short reads were deposited in the NCBI Short Read Archive under the accession number SRP049578.

A BAC library of the globe artichoke was used to search clones harboring the cp genome. A total of 57,600 clones from the same genotype, representing approximately five haploid genome equivalents, were screened by a multidimensional pooling strategy, using cp specific primer pairs (S1 Table). The identified BAC clone was isolated by plasmid DNA extraction and purification with Plasmid Midi Kit (Qiagen, Milan, Italy) following the manufacturer instructions, and finally sent to IGA Technology Services (Udine, Italy) for 250 bp paired-end reads MiSeq (Illumina) sequencing. Short reads were deposited in the NCBI Short Read Archive under the accession number SRR1648410.

Sequence data were analyzed with the CLC Genomics Workbench 6.0.1 (CLC Bio, Aarhus, Denmark), using *de novo* and reference-guided assembling methods, alternatively, with the following parameters: mismatch cost = 2, insertion cost = 3, deletion cost = 3, length fraction = 0.8, similarity fraction = 0.9.

The four junctions between IRs and SSC/LSC were checked by standard PCR amplification with specific primers (S1 Table) and Sanger sequencing.

Gene annotation was carried out with DOGMA (Dual Organellar GenoMe Annotator) [23] to identify coding sequences (cds), rRNAs, and tRNAs using the Plant plastid genetic code and BLAST homology searches. To verify the exact gene and exon boundaries, we compared artichoke annotations with those of lettuce (DQ383816). All tRNA genes were further confirmed with the online tRNAscan-SE 1.21 search server [24].

Codon usage was calculated for all exons of protein-coding genes (pseudogenes were not calculated) with Acua 1.0 [25]. Base composition was evaluated with DNA/RNA Base Composition Calculator (http://www.currentprotocols.com/WileyCDA/CurPro3Tool/toolId-7.html).

Repeated elements in the artichoke cp genome were investigated by means of two web-based programs. The first one, Tandem Repeat Finder [26], was set at 2, 7 and 7 for match, mismatch and indel, respectively. The minimum alignment score and maximum period size were set as 50 and 500, respectively. The second program, REPuter [27], was set with the identity parameter no less than 90% (hamming distance equal to 3). Among dispersed repeats, the minimum repeat size investigated was 30 bp for direct and 20 bp for palindromic, respectively. Overlapping repeats were merged into one repeat motif whenever possible. Microsatellites (SSRs) were predicted using MISA (MIcroSAtellite, http://pgrc.ipk-gatersleben.de/misa/) and the software tool IMEx (Imperfect Microsatellite Extractor) [28]. We identified SSRs as mononucleotide repeats ≥8 bases, dinucleotides ≥10 bases (5 repeats), trinucleotides and tetranucleotides ≥12 bases (4 and 3 repeats, respectively), pentanucleotides ≥15 bp (3 repeats), and exanucleotides ≥18 bp (3 repeats).

### Comparative and phylogenetic analyses of Asteraceae cp genomes and development of barcode markers

Full alignments of nine Asteraceae cp genomes were performed using mVISTA program [29] in Shuffle-LAGAN mode. Genomes retrieved from NCBI were: *Helianthus annuus* (NC007977), *Lactuca sativa* (DQ383816), *Parthenium argentatum* (GU120098), *Guizotia abyssinica* (EU549769), *Jacobaea vulgaris* (HQ234669), *Ageratina adenophora* (NC_015621), *Artemisia frigida* (NC_020607), *Chrisanthemum x morifolium* (JQ362483), and *C*. *cardunculus* var. *scolymus* annotation was used as a reference.

Regions promisingly valuable as phylogenetic markers across the Asteraceae family were investigated with Mega 6 [30] using default parameters (gap opening penalty: 15; gap extension penalty: 6.66; DNA weight matrix: IUB; transition weight: 0.5; negative matrix: off; delay divergent cutoff: 30%). Each alignment was imported in PAUP* 4.0b10 [31] for a phylogenetic analysis using the parsimony criterion. The robustness of every tree was confirmed with 1,000 bootstrap replicates, and the consistency (CI) and retention (RI) indexes were calculated.

For barcoding applications, eight coding regions of the genes *ccsA*, *matK*, *ndhA*, *rbcL*, *accD*, *clpP*, *rps16* and *ycf1* were chosen for primer design with Primer 3 software [32] to obtain amplicons of 800 bp on average. PCR reactions were performed using a 9700 thermal cycler (Applied Biosystems, Foster City, CA) in 10 μl reaction mixtures containing 50 ng template DNA, 0.02 μM forward and reverse primer, 0.2 mM of each dNTP, 1x buffer, 0.4 U Taq DNA polymerase (Life Technologies, Foster City, CA) and 1.5 mM MgCl_2_. Thermal profile for the amplification was 3 min of initial denaturation at 94°C, 35 cycles of 30 sec at 94°C, 30 sec at optimal primer temperature (56°C for all genes, except for *rps16*, at 58°C) and 1 min extension at 72°C, followed by a final 7 min incubation at 72°C. The amplified fragments were checked on 1.5% agarose gel with a 100 bp molecular size standard (Fermentas, Vilnius, Lithuania).

Six cp protein-coding genes (*matk*, *ndhD*, *ndhF*, *ndhI*, *rbcL*, *rpoB*) and the first exon of *rpoC1* were extracted from 69 accessions: 60 from Panero and Funk [10], eight from the NCBI database, and one corresponding to the globe artichoke here described. These species belong to seven Asteraceae subfamilies: Asteroideae, Corymbioideae, Cichorioideae, Gymnarrhenoideae, Pertyoideae, Carduoideae, and Hecastocleidoideae. Extracted sequences were then concatenated through copy and paste and aligned with Fast Statistical Alignment (FSA) [33] web-server, setting gap factor as 1 and model type as Tamura-Nei [34]. All positions containing gaps or missing data were eliminated. Maximum parsimony (MP) analyses were performed with PAUP*4.0b10. Heuristic tree searches were conducted with 10 random-taxon-addition replicates, tree bisection-reconnection (TBR) branch swapping, and with “multrees” option in effect. Non-parametric bootstrap analysis was carried out under 1,000 replicates with TBR branch swapping. Maximum likelihood (ML) analysis was performed with RaxML Blackbox [35] using the Gamma model of rate heterogeneity.

## Results and Discussion

### Chloroplast genome assembly and annotation

Reads from an Illumina partial sequencing of the “Brindisino” globe artichoke nuclear genome were used to assemble the cp genome. To this end, the total reads (33 million) were filtered by aligning them on the cp genome from *L*. *sativa* (DQ383816), chosen for its phyletic proximity to *Cynara* genus. We thus obtained 1,308,860 mapped reads (coverage 643x), covering about 90% of the entire cp genome.

In order to complete the cp sequence, we screened a BAC library obtained from the same genotype used for nuclear genome sequencing. By means of specific primer pairs, a clone harboring the artichoke cp genome was successfully isolated. Illumina BAC sequencing produced longer reads that helped complete the entire cp sequence.

The total amount of reads obtained by the two approaches was merged and assembled using *de novo* and reference-guided methods, separately. The two assemblies produced an almost identical cp sequence, except for six insertion/deletions (indel) events, four insertions and two deletions in the reference-guided assembly compared to the *de novo* one. Subsequent PCR amplifications and Sanger sequencing revealed that the *de novo* assembly was correct five out of six times compared to the reference-guided assembly. Artichoke cp complete sequence was hence adjusted according to these findings. Finally, the four junctions between the IRs and SSC/LSC were confirmed by PCR amplifications and Sanger sequencing.

### Genome organization and gene content

**Fig 1:**
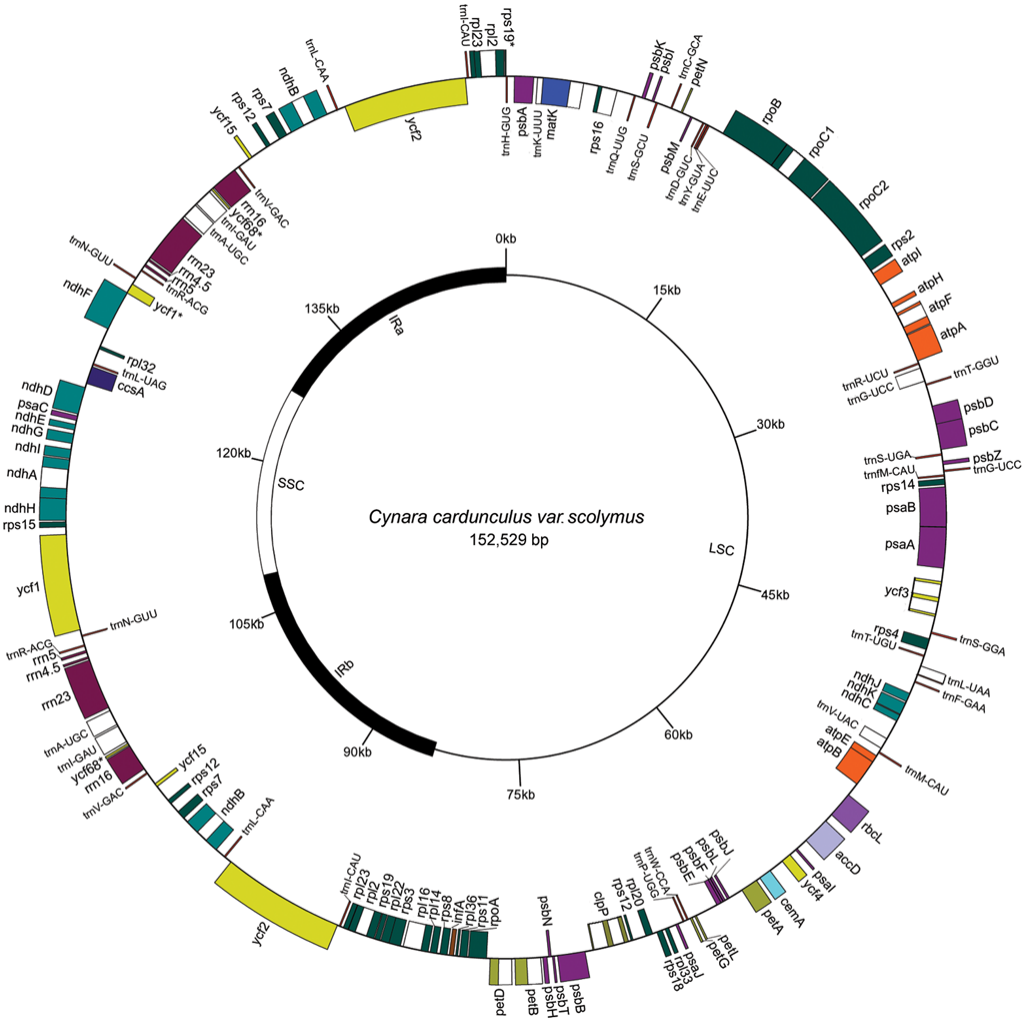
Artichoke cp genome map. Genes shown on the outside of the large circle are transcribed clockwise; genes on the inside are transcribed counterclockwise. Thick lines of the small circle indicate IRs. Pseudogenes are marked with '*'.

The artichoke cp genome is 152,529 bp in length. The canonical quadripartite structure consists of one LSC of 83,578 bp, one SSC of 18,641 bp and a pair of IRs of 25,155 bp each (Fig. 1). This genome contains 114 unique genes, including 30 tRNA, 4 rRNA, and 80 predicted protein-coding genes (Table 1). The tRNA-coding genes represent all the 20 amino acids and are distributed throughout the genome, one in the SSC region, 22 in the LSC region and seven in the IR region. Seven genes coding for tRNA, four rRNA genes and six protein-coding genes (*rpl2*, *rpl23*, *ycf2*, *ndhB*, *rps7*, *ycf15*) are completely duplicated in the IR regions. Therefore, the total number of genes present in the artichoke cp genome is 131 (Fig. 1).

**Table 1:** Genes present in the globe artichoke cp genome.

Category	Gene name
Photosystem I	*psaA*, *B*, *C*, *I*, *J*, *ycf3* ^*a*^, *ycf4*
Photosystem II	*psbA*, *B*, *C*, *D*, *E*, *F*, *H*, *I*, *J*, *K*, *L*, *M*, *N*, *T*, *Z/lhbA*
Cytochrome b6/f	*petA*, *B* ^*b*^, *D* ^*b*^, *G*, *L*, *N*
ATP synthase	*atpA*, *B*, *E*, *F* ^*b*^, *H*, *I*
Rubisco	*rbcL*
NADH oxidoreductase	*ndhA* ^*b*^, *B* ^*b*^ ^,^ ^*c*^, *C*, *D*, *E*, *F*, *G*, *H*, *I*, *J*, *K*
Large subunit ribosomal proteins	*rpl2* ^*b*^ ^,^ ^*c*^, *14*, *16*, *20*, *22*, *23* ^*c*^, *32*, *33*, *36*
Small subunit ribosomal proteins	*rps2*, *3*, *4*, *7* ^*c*^, *8*, *11*, *12* ^*b*^ ^,^ ^*c*^ ^,^ ^*d*^, *14*, *15*, *16* ^*b*^, *18*, *19* ^*c*^ ^,^ ^*e*^
RNAP	*rpoA*, *rpoB*, *C1* ^*a*^, *C2*
Other proteins	*accD*, *ccsA*, *cemA*, *clpP* ^*a*^, *matK*, *infA*
Proteins of unknown function	*ycf1* ^*c*^ ^,^ ^*e*^, *ycf2* ^*c*^, *ycf15* ^*c*^, *ycf68* ^*c*^ ^,^ ^*e*^
Ribosomal RNAs	*rrn23* ^*c*^, *16* ^*c*^, *5* ^*c*^, *4*.*5* ^*c*^
Transfer RNAs	*trnA(UGC)* ^*b*^ ^,^ ^*c*^, *trnC(GCA)*, *D(GUC)*, *E(UUC)*, *F(GAA)*, *G(UCC)*,*G(GCC)*, *H(GUG)*, *I(CAU)* ^*c*^, *I(GAU)* ^*b*^ ^,^ ^*c*^, *K(UUU)* ^*b*^, *L(UAA)* ^*b*^, *L(UAG)*, *L(CAA)* ^*c*^, *fM(CAU)*, *M(CAU)*, *N(GUU)* ^*c*^, *P(UGG)*, *Q(UUG)*, *R(ACG)* ^*c*^, *R(UCU)*, *S(GCU)*, *S(GGA)*, *S(UGA)*, *T(CGU)*, *T(UGU)*, *V(UAC)* ^*b*^, *V(GAC)* ^*c*^, *W(CCA)*, *Y(GUA)*

^a^Gene containing two introns

^b^Gene containing a single intron

^c^Two gene copies in the IRs

^d^Gene divided into two independent transcription units

^e^Pseudogenes

Protein-coding genes make up 49.9% (76,085 nt for 86 genes), tRNAs (2,796 nt for 37 genes) and rRNAs (8,842 nt for eight genes) represent 1.8% and 5.8% of the genome, respectively. The remaining 42.5% are non-coding introns, intergenic spacers, or pseudogenes. We have identified three pseudogenes: *ycf68*, in the IR, contains a premature stop codon in its coding sequence; the remaining two pseudogenes, *ycf1* and *rps19*, are located in the boundary regions between IRb/SSC and IRa/LSC, respectively. The lack of their protein-coding ability is due to a partial gene duplication.

The average AT content of the artichoke cp genome is 62.3%. The AT content of the LSC and SSC regions is 64.2% and 68.6%, respectively, whereas that of the IR regions is 56.9%; these data are congruent to what has been found in other cp genomes, e.g. *Sesamum* and *Camellia* genera [36,37]. The low AT content in the IR regions is due to the reduced presence of AT nucleotides in the four rRNA genes: *rrn16*, *rrn23*, *rrn4*.5, and *rrn5*. The IR regions may help stabilize the cp genome, as evidenced in a group of legumes that have lost a copy of the IR and are subject to more rearrangements compared to those that have not [38].

In the artichoke cp genome there are 18 intron-containing genes (Table 2). Among them, 16 genes (eight protein-coding and six tRNA genes) have a single intron and two genes (*ycf3*, *clpP*) have two introns. Out of the 18 genes with introns, 13 (nine protein-coding and four tRNA genes) are located in the LSC, one protein coding in the SSC and four (two protein coding and two tRNAs) in the IR region. The *trnK-UUU* intron is the largest one (2,530 bp) and includes the *matK* gene. The *rps12* gene is a *trans*-spliced gene: its 5’ end exon is located in the LSC region and the two remaining exons are located in the IR regions. In the *ndhD* and *psbL* genes, we observed that ACG is used as an alternative start codon instead of the canonical ATG. The ACG start codon has been shown to convert to an AUG initiation site as reported in *N*. *tabacum* [39]. One GUG start codon was found in *rps19*. GUG codons have been reported to be more efficient than ACG in initiating translation and have a relative strength varying from 15 to 30% of AUG activity [40].

**Table 2:** Intron containing genes in the globe artichoke cp genome; exon and intron size.

Gene	Region	Exon I (bp)	Intron I (bp)	Exon II (bp)	Intron II (bp)	Exon III (bp)
*rpl16*	LSC	9	1004	399	-	-
*rps16*	LSC	40	854	215	-	-
*rpoC1*	LSC	432	732	1638	-	-
*atpF*	LSC	145	707	410	-	-
*ycf3*	LSC	124	742	230	698	153
*clpP*	LSC	71	626	291	803	229
*petB*	LSC	6	765	642	-	-
rps12*	LSC	243	-	114	-	-
*petD*	LSC	8	705	475	-	-
*rpl2*	IR	391	665	434	-	-
*ndhB*	IR	782	670	751	-	-
*ndhA*	SSC	553	1060	539	-	-
*trnK-UUU*	LSC	37	2530	35	-	-
*trnA-UGC*	IR	38	821	35	-	-
*trnL-UAA*	LSC	37	440	50	-	-
*trnG-UCC*	LSC	47	707	23	-	-
*trnI-GAU*	IR	43	510	35	-	-
*trnV-UAC*	LSC	38	574	38	-	-

* *rps12* gene is subjected to trans-splicing.

A total of 78,891 nt and 26,297 codons represent the coding capacity of 86 protein-coding genes in the artichoke cp genome (S2 Table). Leucine (2,792 codons meaning 10.6% of the total) and cysteine (293 corresponding to 1.1%) are the most and the least abundant amino acids, respectively. The codon usage is biased towards a high representation of A and T at the third codon position, which is similar to the majority of angiosperm cp genomes [41,42].

The whole artichoke cp sequence along with gene annotations was submitted to GenBank (accession number: KM035764).

### Repeat structure and sequence analysis

Repeat regions are considered to play an important role in genome recombination and rearrangement [43,44]. We divided these regions in two categories: direct (D) and palindromic (P) repeats.

With a 100% match criterion in repeat copies, Tandem Repeat Finder (TRF) identified ten sets of repeats longer than 10 bp. With a >90% criterion, TRF detected 12 other sets of repeats giving 22 total sets, nine in cds regions, two in intronic regions, and 11 in intergenic regions (S3 Table).

REPuter allowed us to identify 21 repeats. Six repeats had a 0 hamming distance, that is, a complete identity with each other. We compared the redundant output of REPuter with TRF and checked the tandem repeats; dispersed repeats (direct and palindromic) were analyzed separately. Fifteen palindromic repeats and six direct repeats were identified. Therefore the total number of repeats was 43 and their copy number ranged between 2 and 4 (S3 Table).

We analyzed the length of these repeats: 26 were 10–20 bp, 10 were 21–30 bp, five were 31–40 bp and two were 41–50 bp. Among all the repeats, 50% were in intergenic space regions, 13% in the intronic regions, 34% in the coding regions and 3% in the regions spanning from spacers to gene. The longest repeat was organized in tandem. It measured 45 bp in length and was located in the *ycf1* gene.

Among the coding regions, the richest in repeats was the *ycf1* gene, which contained six repeats: five direct and one palindromic. As reported for other genomes [45,46], the *ycf2* gene was also rich in repeats (four) carrying two direct and two palindromic repeats. It has already been demonstrated that these two coding and divergent regions are often associated with many repeat events [47].

### SSR analysis

Chloroplast SSRs (cpSSRs) are generally short mononucleotide tandem repeats that, when located in the non-coding regions of the cp genome, commonly show intraspecific variation in repeat number [17]. CpSSRs can exhibit high variation within the same species and thus are considered valuable markers for population genetics [48,49] and phylogenetic analyses [50].

**Fig 2:**
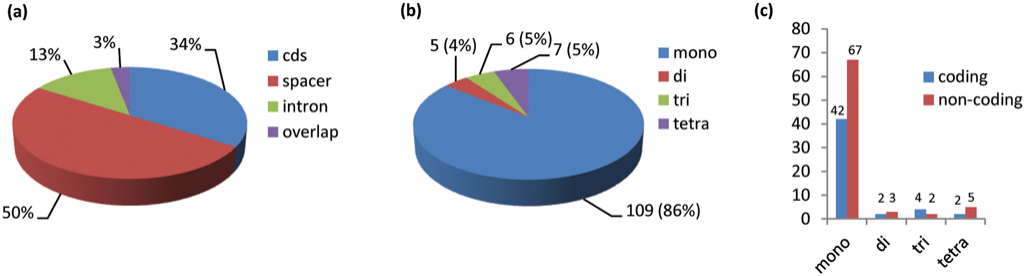
Total repeat and SSR distribution in *C*. *cardunculus* var. *scolymus* chloroplast genome. (a) Repeat distribution among four different regions: coding sequence, intronic sequence, intergenic space region and overlapping region. (b) SSR distribution according to type: mononucleotide, dinucleotide, trinucleotide, and tetranucleotide repeats. SSR number and percentages (in brackets) are provided. (c) SSR type distribution between coding and non-coding regions.

We analyzed SSRs with two programs, IMEx and MISA and obtained comparable results except for 12 SSRs (including four mononucleotides, four dinucleotides and four trinucleotides), which were identified only by IMEX. The total output consisted of 127 repeats: 61% (77 SSRs) in the LSC region, 25% (32 SSRs) in the SSC region, and 14% (18 SSRs) in the IR regions. Furthermore, 46% were in spacer regions, 42% in coding regions, 10% in intronic regions and 2% in pseudogene regions. We found a total of 109 homopolymers corresponding to 86% of the total SSRs, five dinucleotide (4%), six trinucleotide (5%), and seven tetranucleotide (5%) repeats (Fig. 2). Among the 109 mononucleotide repeats, only two belonged to the C/G type while all the others were A/T type; 61.5% of the mononucleotide repeats were in non-coding regions. This higher proportion of poly(A)/(T) relative to poly(G)/(C) has already been reported in Asteraceae [51,52] and other plant families [36,50,53]. The coding cp-regions with the highest number of repeats were *ycf1* with 16 SSRs, followed by *ycf2* with eight SSRs in the two IRs (S4 Table). These results are consistent with those from other species, e.g. *Vigna radiata*, *P*. *argentatum* and *G*. *abyssinica* [41,51,54] emphasizing that the highly variable *ycf1* coding region can represent, also in *Cynara*, an interesting region suitable for phylogenetic studies or DNA barcoding possibly also at low taxonomic levels [55].

### Comparison with other Asteraceae cp genomes, barcode markers and phylogenetic analyses

#### Structural differences: a continuing expansion of IRs in *Cynara*

The artichoke cp genome is the third in size among the nine Asteraceae complete cp genomes (Table 3), smaller than those of *P*. *argentatum* (152,803 bp) and *L*. *sativa* (152,772 bp), and features the largest IR region (25,155 bp). It is important to note that the two *L*. *sativa* genomes available in GenBank (NC_007578 and DQ383816) differ between each other by 6 bp and in the relative orientation of their SSC region. This incongruence can be due to polymorphisms between the strains investigated, to differences in the assembly methods, and/or to sequencing errors. The possible existence of an inverted SSC in Asteraceae genomes is still to be confirmed but cannot be excluded given the nature of the flip-flop mechanism of the inverted repeats [56]. For *Ar*. *frigida*, Liu *et al*. [57] claimed to have observed a totally inverted SSC in their assembly. However, the specific primers they used to validate the presumed inversion event would amplify the SSC no matter its orientation.

**Table 3:** Size comparison among nine cp genomes completely sequenced in the Asteraceae family.

Species	Accession Number	Genome size (bp)	LSC (bp)	SSC (bp)	IR (bp)
*Parthenium argentatum*	NC_013553	152803	84593	18842	24684
*Latuca sativa* cv. *salinas*	DQ383816	152772	84105	18599	25034
*Cynara cardunculus var*. *scolymus*	KM035764	152529	83578	18641	25155
*Guizotia abyssinica*	EU549769	151762	83535	18227	24999
*Helianthus annuus*	NC007977	151104	83530	18308	24633
*Artemisia frigida*	NC_020607	151076	82740	18394	24971
*Chrysanthemum x morifolium*	JQ362483	151033	82780	18347	24953
*Ageratina adenophora*	NC_015621	150698	84829	18359	23755
*Jacobaea vulgaris*	HQ234669	150689	82855	18276	24779

Species are ordered by genome size.

LSC: Large Single-Copy

SSC: Small Single-Copy

IR: Inverted Repeat

**Fig 3:**
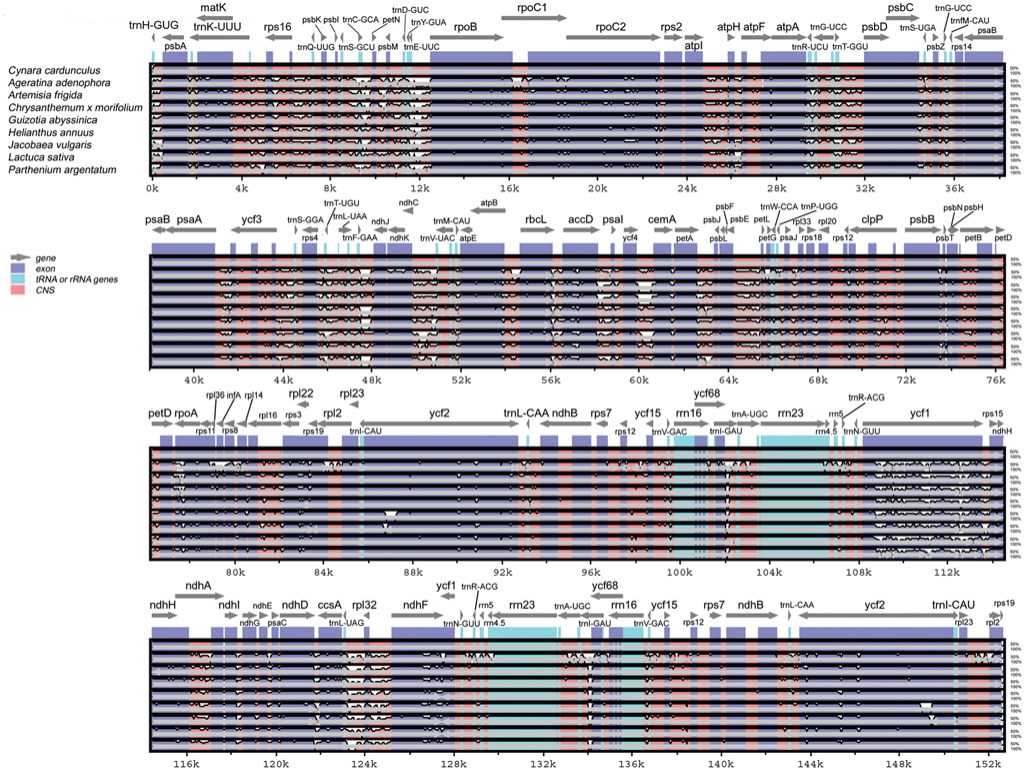
Visualization of alignment of nine Asteraceae cp genome sequences. VISTA-based identity plot showing sequence identity among eight cp genomes already published (see Materials and Methods for accession numbers) and the artichoke cp genome, set as a reference. Sequence identity is shown as a percentage between 50–100% on y-axis. On x-axis, artichoke genes are indicated on top lines, and arrows represent their orientation. Genome regions are distinguished by colors. CNS: conserved non-coding sequences.

A multiple sequence alignment (MSA) was performed among all nine Asteraceae cp genomes sequenced to date, and served as a basis for investigating similarity levels (Fig. 3). In accordance with other angiosperms, the IRs and the coding regions are more conserved than the single-copy and non-coding regions, respectively. The IR regions of cp genome are much conserved in land plants compared to the single copy regions, mainly due to the presence of the rRNA gene group [47]. They only differ in length due to their contraction and expansion at the junction of LSC and SSC. This represents the main cause for size variation in the cp genomes [58,59].

**Fig 4:**
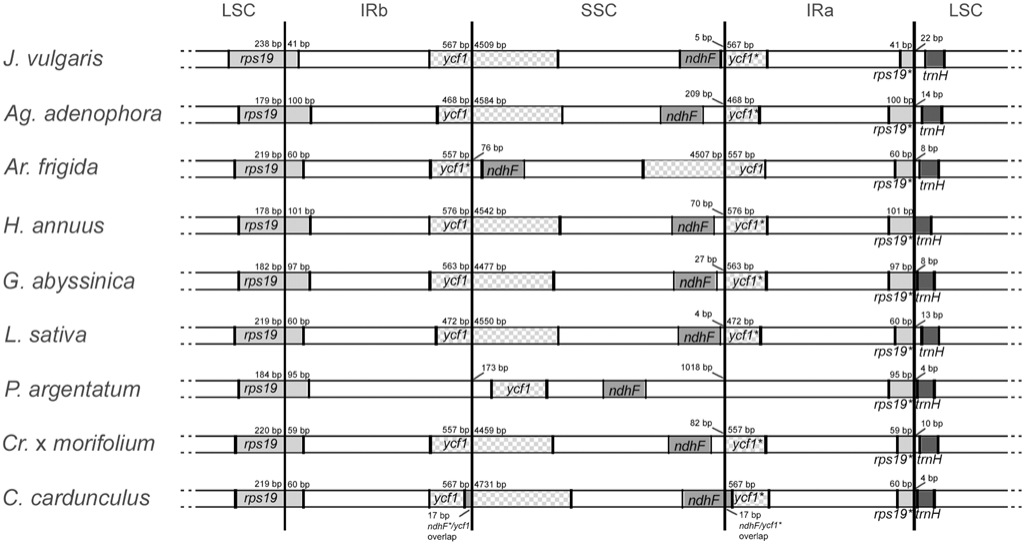
Comparison of the border positions of LSC, SSC, and IR regions among nine sequenced Asteraceae chloroplast genomes. Genes are indicated in boxes and their extensions in the corresponding regions are displayed above boxes.

The IR-LSC/SSC borders with full annotations for the adjacent genes were compared across the nine sequenced Asteraceae cp genomes (Fig. 4). In this comparison, it was necessary to adjust sequence annotations for *J*. *vulgaris*, *Ag*. *adenophora*, *H*. *annuus*, *G*. *abyssinica* and *P*. *argentatum*, so that all sequences started from the first nucleotide after IRa. At the LSC/IRb border, the IRb expanded by 60 bp towards the *rps19* gene in *C*. *cardunculus*, *L*. *sativa* and *Ar*. *frigida*, by 41 bp in *J*. *vulgaris* and by 101 bp in *H*. *annuus*. The same IR expanded by 567 bp in the *ycf1* gene at the IRb/SSC border, both in *C*. *cardunculus* and *J*. *vulgaris*. At this position, the smallest and biggest expansions occur in *Ag*. *adenophora* (468 bp) and *H*. *annuus* (576 bp), respectively. In seven out of nine species, the complete *ycf1* gene spans across IRb and SSC and appears as a pseudogene in the IRa region. The contrary happens in *Ar*. *frigid*a and its inverted SSC [57]; in *P*. *argentatum*, the *ycf1* gene is entirely located in the SSC region.

The *ndhF* gene in *C*. *cardunculus* overlaps the SSC/IRa border by 17 bp, revealing an expansion of the IR compared to the other Asteraceae cp genomes sequenced so far. In this way, 17 bp at the 3’ end of *ndhF* gene are overlapping with *ycf1* gene at the IRb/SSC border and with *ycf1* pseudogene at the SSC/IRa border. In all other eight species, the same *ndhF* gene is entirely located in the SSC region varying only in distance from the SSC/IRa border. This distance is only 4 bp and 5 bp in *L*. *sativa* and *J*. *vulgaris*, respectively, whereas in *P*. *argentatum* it is 1,018 bp. In *Ar*. *frigida* the same gene is 76 bp distant from the IRb/SSC border, because of its inverted SSC region.

#### Highly informative regions and barcoding perspectives

Based on MSA of the nine Asteraceae cp genomes (Fig. 3), we focused on coding regions and retrieved the most promising sequences suitable for the development of reliable molecular markers in the Asteraceae family. The intergenic sequences may not be appropriate for phylogenetic analyses at the family level due to their high variation and a lack of high quality alignments [60] and thus should rather be used at a lower taxonomic rank.

After aligning separately the selected coding regions and investigating the parsimony-informative ratios, we analyzed the most divergent regions (Table 4). *Ycf1* and *rps16* displayed the highest percentage of parsimony-informative characters (8.6% and 6.1% respectively), while the other coding regions analyzed (*ccsA*, *rbcL*, *ndhA*, *ndhF*, *matK*, *clpP*, *accD*, *petD*, *petB* and *rpoC1*) showed an interesting parsimony-informative ratio ranging from 3.9% to 5.4%. With this analysis, we confirmed the informative values for well-known regions previously adopted for the Asteraceae family (i.e. *rbcL*, *rps16*, *ndhF*, *matK*), or recently observed, such as *rpoC1*, *ycf1* and *clpP* genes [60,52]. Moreover, the genes *accD*, *ccsA*, *ndhA*, *petB* and *petD* were identified in this work as highly parsimony-informative regions and thus can be considered in future phylogenetic studies in this family. *Ycf1*, *clpP and accD* are essential genes for cell survival and plant development in some taxa, but not in others [61,62,63]. *Rps16*, the gene coding for the ribosomal protein S16, appears non-functional or lost in several plant lineages, e.g. *Medicago truncatula*, *Phaseolus vulgaris*, *V*. *radiata* and the *Populus* genus [64]. Due to their pivotal role, these genes can be substituted by nuclear-encoded versions when the cp forms are not functional or lacking [65].

**Table 4:** Coding regions and their parsimony-informative rate.

No.	Region	Length	Aligned length	Conserved sites	No. Pars. uninf.	Pars. inf.	Pars.inf. %	C.I.	R.I.
1	*accD*	1530	1610	1414	130	66	4.10	0.91	0.79
2	*ccsA*	969	975	859	63	53	5.44	0.88	0.79
3	*cemA*	690	690	632	40	18	2.61	0.94	0.85
4	*clpP*	2020	2127	1894	144	89	4.18	0.9	0.81
5	*matK*	1521	1527	1342	119	66	4.32	0.94	0.88
6	*ndhA*	2152	2307	2015	190	102	4.42	0.90	0.77
7	*ndhI*	501	501	470	18	13	2.59	0.92	0.87
8	*ndhK*	678	678	592	75	11	1.62	0.95	0.67
9	*petB*	1413	1444	1285	103	56	3.88	0.88	0.74
10	*petD*	1188	1269	1160	58	51	4.02	0.92	0.88
11	*rbcL*	1434	1458	1340	52	66	4.53	0.78	0.67
12	*rpoA*	1008	1014	896	93	25	2.47	0.92	0.72
13	*rpoC1*	2802	2918	2030	778	110	3.77	0.94	0.66
14	*rpoC2*	4158	4176	3763	288	125	2.99	0.92	0.79
15	*rps16*	1109	1238	1034	129	75	6.06	0.92	0.82
16	*ycf1*	5304	5568	3585	1505	478	8.58	0.89	0.60
17	*ndhD*	1503	1539	1420	79	40	2.60	0.85	0.64
18	*ndhF*	2250	2260	1983	173	104	4.60	0.89	0.78
19	*rpoB*	3183	3183	2965	140	76	2.39	0.91	0.82

Length: refers to sequence length in *Cynara cardunculus* var. *scolymus*

Aligned length: refers to the alignment of nine Asteraceae considered in the comparative analysis (see Materials and Methods)

Pars.: parsimony

Uninf. uninformative

Inf.: informative

C.I.: consistency index

R.I.: retention index

The presence of intronic sequences in both *ndhA* and *rps16* genes contributes to the divergence at these two loci. *MatK* gene has been shown to have a high evolutionary rate and a suitable length for barcoding applications. *RbcL* is a good candidate for DNA barcoding in plants at the family and genus level too, since it can be easily amplified and sequenced in most land plants. Nevertheless, it shows a slow evolutionary rate and a lower divergence compared to the other plastid genes in flowering plants [66]. Thanks to their complementary features, *matK* and *rbcL* have been recommended by the Consortium for the Barcode of Life (CBOL) Plant Working Group in combination as multi-locus DNA barcodes in plants [20].

In order to propose possible barcode regions for the Asteraceae family, we focused on eight of the genes described above, which displayed a rate of informativity above 4%: *ycf1*, *rps16*, *ccsA*, *rbcL*, *ndhA*, *matK*, *clpP* and *accD* (Table 4). Based on MSA among the nine species completely sequenced, we designed “universal” primer pairs (S5 Table) which can be used in the whole Asteraceae family. In order to test their efficiency, we amplified a group of species (*L*. *serriola*, *Matricaria chamomilla*, *Gerbera hybrida*, *Cr*. x *morifolium*, *H*. *annuus* and *C*. *cardunculus*). These species are representatives of the four major Asteraceae subfamilies (Asteroideae, Cichorioideae, Carduoideae and Multisioideae) which are estimated to include 99% of the Asteraceae species [1]. We obtained 100% successful amplifications (S1 Fig.) with specific products of the expected sizes, suggesting that these primer pairs can be useful for species barcoding within the Asteraceae family.

#### Phylogenetic relationships within Asteraceae

**Fig 5:**
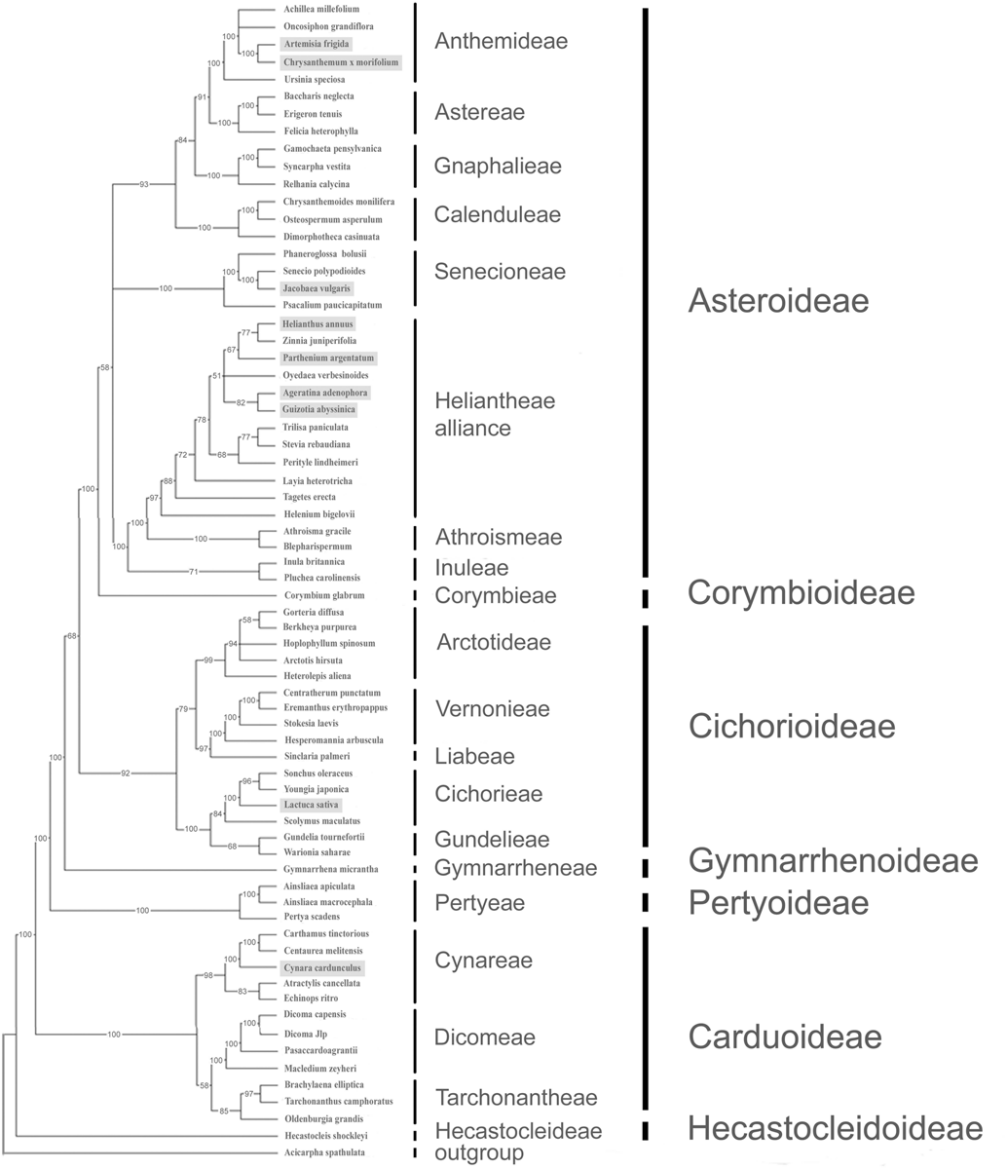
Phylogenetic tree based on maximum parsimony of 69 accessions belonging to the Asteraceae family. Seven coding regions were used: *matk*, *ndhD*, *ndhF*, *ndhI*, *rbcL*, *rpoB* and the first exon of *rpoC1*, for a total of 1,811 parsimony-informative characters. Sequences from *C*. *cardunculus* were obtained from this work. Bootstrap values for each node were set greater than 50%. Species for which the complete cp genome is available are shaded.

Asteraceae is one of the largest families in the plant kingdom. Several studies have analyzed the phylogenetic relationships in this family based on cp sequences. One of the most comprehensive analyses included 108 taxa and was based on ten cp regions, seven of which were coding genes, and the remaining ones noncoding sequences [10]. However, this study did not involve the genus *Cynara* and most of the species for which cp genome has been completely sequenced. Therefore, in order to place new species in the Asteraceae metatree, we selected 60 taxa from that work, belonging to the main Asteraceae subfamilies, and added the nine completely sequenced cp genomes, including *C*. *cardunculus*. For this purpose, we retrieved six intronless genes (*matk*, *ndhD*, *ndhF*, *ndhI*, *rbcL*, *rpoB*) and the first exon of *rpoC1*. Gene sequences for each single *taxon* were concatenated and then aligned. Total alignment was 13,875 bp in length, comprising 10,491 constant characters, 1,573 singleton characters and 1,811 parsimony-informative characters. Maximum Parsimony and ML analyses were performed using *Acicarpha spatulata* as outgroup. The two trees obtained displayed comparable topologies and only slightly better bootstrap values were obtained with ML method. With MP analysis, a phylogenetic tree of 7,380 total length was obtained (Fig. 5), whereas ML delivered a tree with a sum of branch lengths of SBL = 0.7888 (S2 Fig.). The consensus MP tree, displaying bootstrap values higher than 70% in almost all nodes, was highly comparable with the MP tree obtained by Panero and Funk [10], even though in our analysis we did not include the non-coding cp regions *trn*L-*trn*F, 23S-*trn*A, and *trn*K partial intron. Moreover, we added the species with newly sequenced cp genomes, placing them in the Asteraceae phylogenetic tree.

Within the Asteroideae subfamily, our MP tree showed *Cr*. x *morifolium* grouping with *Ar*. *frigida* within Anthemideae tribe. As expected, *Jacobea vulgaris* clustered in the Senecioneae tribe. However, the relationship between this tribe and other tribes of Asteroideae was not solved. This result is in agreement with those obtained by Panero and Funk [10] who found the position of Senecioneae equivocal. *Helianthus annuus*, *P*. *argentatum*, *G*. *abyssinica*, and *Ag*. *adenophora* grouped in the Heliantheae alliance. The Asteroideae subfamily is sister to Cichorioideae subfamily, including *L*. *sativa* in the Cichorieae tribe. Both Asteroideae and Cichorioideae are related to the Carduoideae subfamily; here the previous tricotomy among Tarchonantheae, Dicomeae, and Cynareae [10] was solved by grouping Dicomeae and Tarchonantheae tribes, although with low bootstrap support (58% and 64% in MP and ML trees, respectively). Taxa within the Cynareae tribe form two groups. The first one is composed of *Carthamus tinctorius* and *Centaurea melitensis*, and, to a higher level, *C*. *cardunculus*. The second group includes *Atractylis cancellata* and *Echinops ritro*. The phylogenetic results on Cynareae tribe are consistent with those observed in more detailed studies specific to Cardueae (Cynareae) based on morphological [67] and molecular evidence [11], although a lower number of species and genera was considered in our analysis.

## Conclusions

The *C*. *cardunculus* chloroplast genome represents the first complete sequence from the large Carduoideae subfamily, within the widespread family of Asteraceae. The comparison with the eight other Asteraceae complete genomes sequenced so far demonstrated that the artichoke cp genome is well conserved in gene content and order but that it also features a relevant number of simple sequence repeats, which could be further explored for population studies within *Cynara* genus. The most parsimony-informative regions identified in this study are of potential interest for future phylogenetic studies of the Asteraceae and may serve as a solid resource for barcoding applications.

## Supporting Information

S1 Fig Test results for the eight barcoding-candidate primer pairs designed for selected parsimony-informative regions. 1: *Cr*. *x morifolium*; 2: *M*. *chamomilla*; 3: *Gerbera hybrida*; 4: *L*. *serriola*; 5: *H*. *annuus*; 6: *C*. *cardunculus*. Left lane in each gel: 100 bp DNA ladder.
(TIF)

S2 Fig Phylogenetic tree based on maximum likelihood of 69 accessions belonging to the Asteraceae family. Seven coding regions were used: *matk*, *ndhD*, *ndhF*, *ndhI*, *rbcL*, *rpoB* and the first exon of *rpoC1*. The analysis was performed using RaxML Blackbox with the Gamma model of rate heterogeneity. Bootstrap support values were set greater than 50%.
(TIF)

S1 Table Primer pairs used for BAC identification and junction validation. The name of primers refer to the cp regions amplified.
(DOCX)

S2 Table Codon-anticodon recognition pattern and codon usage for *Cynara cardunculus* var. *scolymus* chloroplast genome. (DOCX)

S3 Table Direct and palindromic repeats in *C*. *cardunculus* var. *scolymus* cp genome. (DOCX)

S4 Table SSRs found in artichoke cp genome. (DOCX)

S5 Table Barcoding primer pair candidates. Sequences were designed on the most parsimony-informative coding regions revealed by the multialignment of the nine Asteraceae complete cp genomes.
(DOCX)

## References

[1] Bremer K. Asteraceae: cladistics and classification. Portland, Oregon, USA: Timber Press; 1994.

[2] Rottenberg A, Zohary D. The wild ancestry of the cultivated artichoke. Genet Resour Crop Evol. 1996; 43: 53–58.

[3] Sonnante G, Pignone D, Hammer K. The domestication of artichoke and cardoon: from Roman times to the genomic age. Ann Bot. 2007; 100: 1095–1100. 17611191 10.1093/aob/mcm127 PMC2759203

[4] Gebhardt R. Antioxidative and protective properties of extracts from leaves of the artichoke (*Cynara scolymus* L.) against hydroperoxide-induced oxidative stress in cultured rat hepatocytes. Toxicol Appl Pharmacol. 1997; 144: 279–286. 9194411 10.1006/taap.1997.8130

[5] Sonnante G, D’Amore R, Blanco E, Pierri CL, De Palma M, Luo J, et al Novel hydroxycinnamoyl-CoenzymeAquinate transferase genes from artichoke are involved in the synthesis of chlorogenic acid. Plant Physiol. 2010; 153: 1–15. 10.1104/pp.109.150144 PMC2899911 20431089

[6] Negro D, Montesano V, Grieco S, Crupi P, Sarli G, De Lisi A, et al Polyphenol compounds in artichoke plant tissues and varieties. J Food Sci. 2012; 77: C244–252. 10.1111/j.1750-3841.2011.02531.x 22251096

[7] Kraft K. Artichoke leaf extract: recent findings reflecting effects on lipid metabolism, liver and gastrointestinal tracts. Phytomedicine 1997; 4: 369–378. 10.1016/S0944-7113(97)80049-9 23195590

[8] Gatto A, De Paola D, De Bagnoli F, Vendramin GG, Sonnante G. Population structure of *Cynara cardunculus* complex and the origin of the conspecific crops artichoke and cardoon. Ann Bot. 2013; 112: 855–865. 10.1093/aob/mct150 23877076 PMC3747803

[9] Calabrese N, Carito A, Boari F, Cantore V, De Palma E, Damato G. Agronomical evaluation of artichoke cultivar propagated by seed. Acta Hortic. 2011; 942: 153–158.

[10] Panero JL, Funk VA. The value of sampling anomalous taxa in phylogenetic studies: Major clades of the Asteraceae revealed. Mol Phylogenet Evol. 2008; 47: 757–782. 10.1016/j.ympev.2008.02.011 18375151

[11] Barres L, Sanmartín I, Anderson CL, Susanna A, Buerki S, Galbany-Casals M, et al Reconstructing the evolution and biogeographic history of tribe Cardueae (Compositae). Am J Bot. 2013; 100: 867–882. 10.3732/ajb.1200058 23624927

[12] Dyall SD, Brown MT, Johnson PJ. Ancient invasions: from endosymbionts to organelles. Science 2004; 304: 253–257. 15073369 10.1126/science.1094884

[13] Odintsova MS, Yurina NP. Chloroplast genomics of land plants and algae In: Giardi MT, Piletska EV, editors. Biotechnological applications of photosynthetic proteins: biochips, biosensors and biodevices. Georgetown, TX, USA: Landes Bioscience/Eurekah; 2006.

[14] Olmstead RG, Palmer JD. Chloroplast DNA systematic: a review of methods and data analysis. Am J Bot. 1994; 81: 1205–1224.

[15] Jansen RK, Raubeson LA, Boore JL, dePamphilis CW, Chumley TW, Haberle RC, et al Methods for obtaining and analyzing whole chloroplast genome sequences. Methods Enzymol. 2005; 395: 348–384. 15865976 10.1016/S0076-6879(05)95020-9

[16] Wolfe KH, Li WH, Sharp PM. Rates of nucleotide substitution vary greatly among plant mitochondrial, chloroplast, and nuclear DNAs. Proc Natl Acad Sci USA. 1987; 84: 9054–9058. 3480529 10.1073/pnas.84.24.9054 PMC299690

[17] Provan J, Powell W, Hollingsworth PM. Chloroplast microsatellites: new tools for studies in plant ecology and evolution. Trends Ecol Evol. 2001; 16: 142–147. 11179578 10.1016/s0169-5347(00)02097-8

[18] Ravi V, Khurana JP, Tyagi AK, Khurana P. An update on chloroplast genome. Plant Syst Evol. 2008; 271: 101–122.

[19] Ahmed I, Matthews PJ, Biggs PJ, Naeem M, McLenachan PA, Lockhart PJ. Identification of chloroplast genome loci suitable for high resolution phylogeographic studies of *Colocasia esculenta* (L.) Schott (Araceae) and closely related taxa. Mol Ecol Resour. 2013; 13: 929–937. 10.1111/1755-0998.12128 23718317

[20] Li X, Yang Y, Henry RJ, Rossetto M, Wang Y, Chen S. Plant DNA barcoding: from gene to genome. Biol Rev. 2015; 90: 157–166. 10.1111/brv.12104 24666563

[21] Shinozaki K, Ohme M, Tanaka M, Wakasugi T, Hayashida N, Matsubayashi T, et al The complete nucleotide sequence of the tobacco chloroplast genome: its gene organization and expression. EMBO J. 1986; 5: 2043–2049. 16453699 10.1002/j.1460-2075.1986.tb04464.x PMC1167080

[22] Sonnante G, De Paolis A, Lattanzio V, Perrino P. Genetic variation in wild and cultivated artichoke revealed by RAPD markers. Genet Resour Crop Evol. 2002; 49:247–252.

[23] Wyman SK, Jansen RK, Boore JL. Automatic annotation of organellar genomes with DOGMA. Bioinformatics. 2004; 20: 3252–3255. 15180927 10.1093/bioinformatics/bth352

[24] Lowe TM, Eddy SR. tRNAscan-SE: a program for improved detection of transfer RNA genes in genomic sequence. Nucleic Acids Res. 1997; 25: 955–964. 9023104 10.1093/nar/25.5.955 PMC146525

[25] Umashankar V, Arunkumar V, Dorairaj S. ACUA: A software tool for automated codon usage analysis. Bioinformation. 2007; 2: 62–63. 18188422 10.6026/97320630002062 PMC2174420

[26] Benson G. Tandem repeats finder: a program to analyze DNA sequences. Nucleic Acids Res. 1999; 27: 573–580. 9862982 10.1093/nar/27.2.573 PMC148217

[27] Kurtz S, Choudhuri JV, Ohlebusch E, Schleiermacher C, Stoye J, Giegerich R, et al REPuter: the manifold applications of repeat analysis on a genomic scale. Nucleic Acids Res. 2001; 29: 4633–4642. 11713313 10.1093/nar/29.22.4633 PMC92531

[28] Mudunuri SB, Nagarajaram HA. IMEx: imperfect microsatellite extractor. Bioinformatics. 2007; 23: 1181–7. 17379689 10.1093/bioinformatics/btm097

[29] Frazer KA, Pachter L, Poliakov A, Rubin EM, Dubchak I. VISTA: computational tools for comparative genomics. Nucleic Acids Res. 2004; 32: W273–W279. 15215394 10.1093/nar/gkh458 PMC441596

[30] Tamura K, Stecher G, Peterson D, Filipski A, Kumar S. MEGA6: Molecular Evolutionary Genetics Analysis Version 6.0. Mol Biol Evol. 2013; 30: 2725–2729. 10.1093/molbev/mst197 24132122 PMC3840312

[31] Swofford DL. PAUP*: Phylogenetic Analysis Using Parsimony (*and other methods), Version 4. Sunderland, MA: Sinauer Associates; 2003.

[32] Untergrasser A, Cutcutache I, Koressaar T, Ye J, Faircloth BC, Remm M, et al Primer3—new capabilities and interfaces. Nucleic Acids Res. 2012; 40: e115 22730293 10.1093/nar/gks596 PMC3424584

[33] Bradley RK, Roberts A, Smoot M, Juvekar S, Do J, Dewey C, et al Fast statistical alignment. PLoS Comput Biol. 2009; 5: e1000392 10.1371/journal.pcbi.1000392 19478997 PMC2684580

[34] Tamura K, Nei M. Estimation of the number of nucleotide substitutions in the control region of mitochondrial DNA in humans and chimpanzees. Mol Biol Evol. 1993; 10: 512–526. 8336541 10.1093/oxfordjournals.molbev.a040023

[35] Stamatakis A, Hoover P, Rougemont J. A rapid bootstrap algorithm for the RAxML web servers. Syst Biol. 2008; 57: 758–771. 10.1080/10635150802429642 18853362

[36] Yi D, Kim K. Complete chloroplast genome sequences of important oilseed crop *Sesamum indicum* L. PLoS ONE. 2012; 7: e35872 10.1371/journal.pone.0035872 22606240 PMC3351433

[37] Yang JB, Yang SX, Li HT, Yang J, Li DZ. Comparative chloroplast genomes of *Camellia* species. PLoS ONE. 2013; 8: e73053 10.1371/journal.pone.0073053 24009730 PMC3751842

[38] Palmer JD, Thompson WF. Chloroplast DNA rearrangements are more frequent when a large inverted repeat sequence is lost. Cell. 1982; 29: 537–550. 6288261 10.1016/0092-8674(82)90170-2

[39] Sasaki T, Yukawa Y, Miyamoto T, Obokata J, Sugiura M. Identification of RNA editing sites in chloroplast transcripts from the maternal and paternal progenitors of tobacco (*Nicotiana tabacum*): comparative analysis shows the involvement of distinct trans-factors for *ndhB* editing. Mol Biol Evol. 2003; 20: 1028–1035. 12716996 10.1093/molbev/msg098

[40] Rohde W, Gramstat A, Schmitz J, Tacke E, Prϋfer D. Plant viruses as model systems for the study of non-canonical translation mechanisms in higher plants. J Gen Virol. 1994; 75: 2141–2149. 8077913 10.1099/0022-1317-75-9-2141

[41] Tangphatsornruang S, Sangsrakru D, Chanprasert J, Uthaipaisanwong P, Yoocha T, Jomchai N, et al The chloroplast genome sequence of mungbean (*Vigna radiata*) determined by high-throughput pyrosequencing: structural organization and phylogenetic relationships. DNA Res. 2010; 17: 1–22. 10.1093/dnares/dsp024 20007682 PMC2818187

[42] Qian J, Song J, Gao H, Zhu Y, Xu J, Pang X, et al The complete chloroplast genome sequence of the medicinal plant *Salvia miltiorrhiza* . PLoS ONE. 2013; 8: e57607 10.1371/journal.pone.0057607 23460883 PMC3584094

[43] Ogihara Y, Terachi T, Sasakuma T. Intramolecular recombination of chloroplast genome mediated by short direct-repeat sequences in wheat species. Proc Natl Acad Sci USA. 1988; 85: 8573–8577. 3186748 10.1073/pnas.85.22.8573 PMC282501

[44] Milligan BG, Hampton JN, Palmer JD. Dispersed repeats and structural reorganization in subclover chloroplast DNA. Mol Biol Evol. 1989; 6: 355–368. 2615639 10.1093/oxfordjournals.molbev.a040558

[45] Bausher MG, Singh ND, Lee SB, Jansen RK, Daniell H. The complete chloroplast genome sequence of Citrus sinensis (L.) Osbeck var ‘ridge pineapple’: organization and phylogenetic relationships to other angiosperms. BMC Plant Biol. 2006; 6: 21 17010212 10.1186/1471-2229-6-21 PMC1599732

[46] Jansen RK, Kaittanis C, Saski C, Lee SB, Tomkins J, Alverson AJ, et al Phylogenetic analyses of *Vitis* (Vitaceae) based on complete chloroplast genome sequences: effects of taxon sampling and phylogenetic methods on resolving relationships among rosids. BMC Evol Biol. 2006; 6: 32 16603088 10.1186/1471-2148-6-32 PMC1479384

[47] Li R, Ma PF, Wen J, Yi TS. Complete Sequencing of Five Araliaceae Chloroplast Genomes and the Phylogenetic Implications. PLoS ONE. 2013; 8: e78568 10.1371/journal.pone.0078568 24205264 PMC3799623

[48] Grassi F, Labra M, Scienza A, Imazio S. Chloroplast SSR markers to assess DNA diversity in wild and cultivated grapevines. Vitis. 2002; 41: 157–158.

[49] Timme RE, Kuehl JV, Boore JL, Jansen RK. A comparative analysis of the *Lactuca* and *Helianthus* (Asteraceae) plastid genomes: identification of divergent regions and categorization of shared repeats. Am J Bot. 2007; 94: 302–312. 10.3732/ajb.94.3.302 21636403

[50] Melotto-Passarin D, Tambarussi E, Dressano K, De Martin V, Carrer H. Characterization of chloroplast DNA microsatellites from *Saccharum* spp and related species. Genet Mol Res. 2011; 10: 2024–2033. 10.4238/vol10-3gmr1019 21948764

[51] Kumar S, Hahn FM, McMahan CM, Cornish K, Whalen MC. Comparative analysis of the complete sequence of the plastid genome of *Parthenium argentatum* and identification of DNA barcodes to differentiate *Parthenium* species and lines. BMC Plant Biol. 2009; 9: 131 10.1186/1471-2229-9-131 19917140 PMC2784773

[52] Nie X, Lv S, Zhang Y, Du X, Wang L, Biradar SS et al. Complete chloroplast genome sequence of a major invasive species, crofton weed (*Ageratina adenophora*). PLoS ONE. 2012; 7: e36869 10.1371/journal.pone.0036869 22606302 PMC3350484

[53] Martin G, Baurens FC, Cardi C, Aury JM, D’Hont A. The complete chloroplast genome of banana (*Musa acuminata*, Zingiberales): insight into plastid monocotyledon evolution. PLoS ONE. 2013; 8: e67350 10.1371/journal.pone.0067350 23840670 PMC3696114

[54] Dempewolf H, Kane NC, Ostevik KL, Geleta M, Barker MS, Lai Z, et al Establishing genomic tools and resources for *Guizotia abyssinica* (L.f.) Cass.-the development of a library of expressed sequence tags, microsatellite loci, and the sequencing of its chloroplast genome. Mol Ecol Resour. 2010; 10: 1048–1058. 10.1111/j.1755-0998.2010.02859.x 21565115

[55] Dong W, Liu J, Yu J, Wang L, Zhou S. Highly variable chloroplast markers for evaluating plant phylogeny at low taxonomic levels and for DNA barcoding. PLoS ONE. 2012; 7(4): e35071 10.1371/journal.pone.0035071 22511980 PMC3325284

[56] Martin GE, Rousseau-Gueutin M, Cordonnier S, Lima O, Michon-Coudouel S, Naquin D, et al The first complete chloroplast genome of the genistoid legume *Lupinus Luteus*: evidence for a novel major lineage-specific rearrangement and new insights regarding plastome evolution in the legume family. Ann Bot. 2014; 113: 1197–1210. 10.1093/aob/mcu050 24769537 PMC4030815

[57] Liu Y, Huo N, Dong L, Wang Y, Zhang S, Young HA, et al Complete chloroplast genome sequences of Mongolia medicine *Artemisia frigida* and phylogenetic relationships with other plants. PLoS ONE. 2013; 8: e57533 10.1371/journal.pone.0057533 23460871 PMC3583863

[58] Chung HJ, Jong DJ, Park HW. The complete chloroplast genome sequences of *Solanum tuberosum* and comparative analysis with Solanaceae species identified the presence of a 241-bp deletion in cultivated potato chloroplast DNA sequence. Plant Cell Rep. 2006; 25: 1369–1379. 16835751 10.1007/s00299-006-0196-4

[59] Wolf PG, Roper JM, Duffy AM. The evolution of chloroplast genome structure in ferns. Genome. 2010; 53: 731–738. 10.1139/g10-061 20924422

[60] Doorduin L, Gravendeel B, Lammers Y, Ariyurek Y, Chin-A-Woeng T, Vrieling K. The complete chloroplast genome of 17 individuals of pest species *Jacobaea vulgaris*: SNPs, microsatellites and barcoding markers for population and phylogenetic studies. DNA Res. 2011; 18: 93–105. 10.1093/dnares/dsr002 21444340 PMC3077038

[61] Drescher A, Ruf S, Calsa TJ, Carrer H, Bock R. The two largest chloroplast genome-encoded open reading frames of higher plants are essential genes. Plant J. 2000; 22: 97–104. 10792825 10.1046/j.1365-313x.2000.00722.x

[62] Kuroda H, Maliga P. The plastid *clpP1* protease gene is essential for plant development. Nature. 2003; 425: 86–89. 12955146 10.1038/nature01909

[63] Kode V, Mudd EA, Iamtham S, Day A. The tobacco plastid *accD* gene is essential and is required for leaf development. Plant J. 2005; 44: 237–244. 16212603 10.1111/j.1365-313X.2005.02533.x

[64] Ueda M, Nishikawa T, Fujimoto M, Takanashi H, Arimura S, Tsutsumi N, et al Substitution of the gene for chloroplast *RPS16* was assisted by generation of a dual targeting signal. Mol Biol Evol. 2008; 25: 1566–1575. 10.1093/molbev/msn102 18453549

[65] Martínez-Alberola F, del Campo EM, Lázaro-Gimeno D, Mezquita-Claramonte S, Molins A, Mateu-Andrés I, et al Balanced gene losses, duplications and intensive rearrangements led to an unusual regularly sized genome in *Arbutus unedo* chloroplasts. PLoS ONE. 2013; 8: e79685 10.1371/journal.pone.0079685 24260278 PMC3832540

[66] Kress WJ, Erickson DL. A two-locus global DNA barcode for land plants: the coding *rbcL* gene complements the non-coding *trnH–psbA* spacer region. PLoS ONE. 2007; 2: e508 17551588 10.1371/journal.pone.0000508 PMC1876818

[67] Ortiz S, Bonifacino JM, Crisci JV, Funk VA, Hansen HV, Hind DJN, et al The basal grade of the Compositae: Mutisieae (sensu Cabrera) and Carduoideae In: Funk VA, Susanna A, Stuessy TF, Bayer RJ, editors. Systematics, evolution, and biogeography of Compositae. Vienna, Austria: International Association for Plant Taxonomy; 2009 pp. 193–213.

