# Supplementary figures and images for: Complete Chloroplast Genome of the Multifunctional Crop Globe Artichoke and Comparison with Other Asteraceae
Source: PLoS One. 2015 Mar 16;10(3):e0120589. doi: 10.1371/journal.pone.0120589 (PMC4361619; doi:10.1371/journal.pone.0120589)

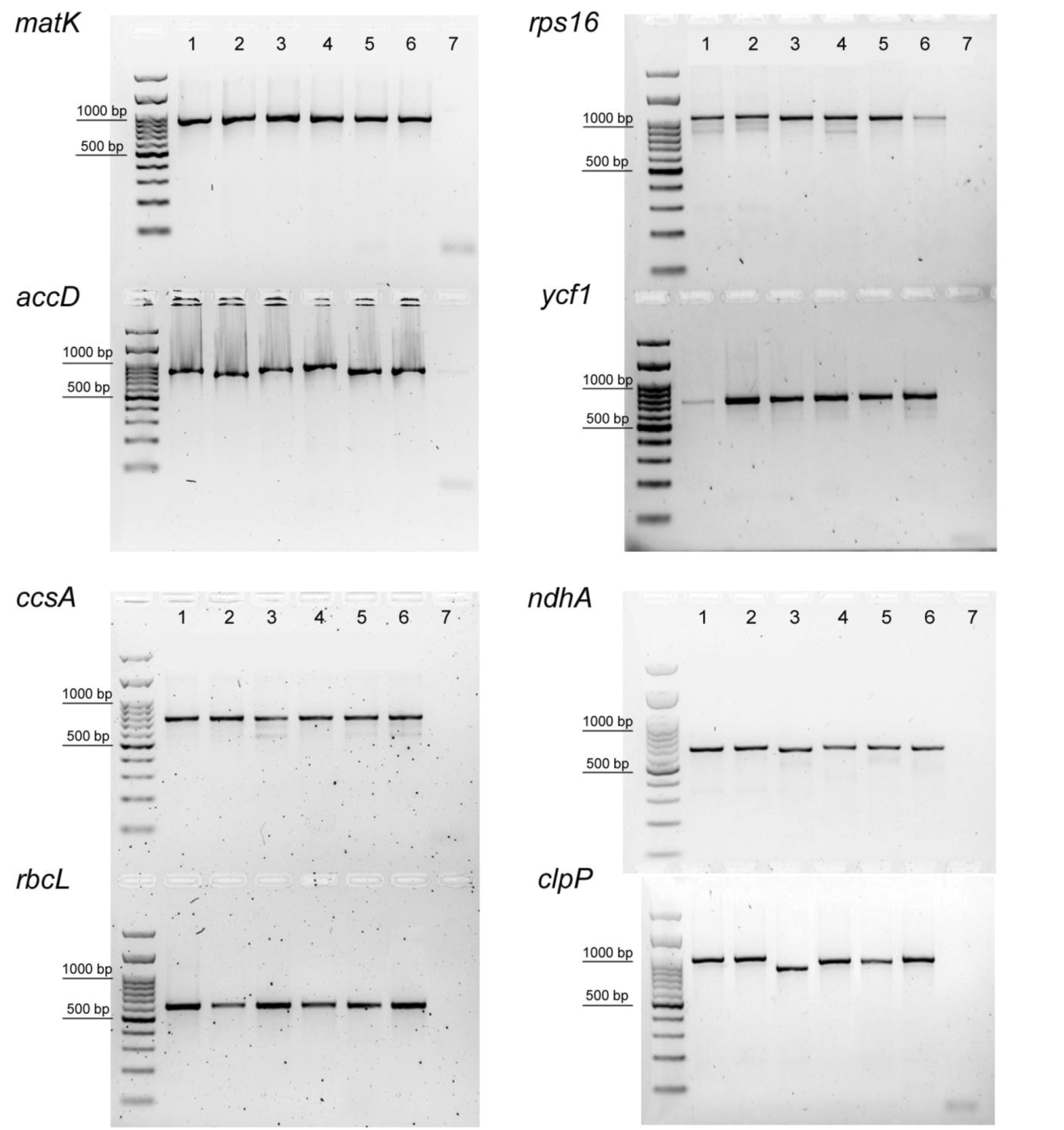

Supplement: S1 Fig — 1: Cr. x morifolium; 2: M. chamomilla; 3: Gerbera hybrida; 4: L. serriola; 5: H. annuus; 6: C. cardunculus. Left lane in each gel: 100 bp DNA ladder. (TIF) [file pone.0120589.s001.tif]

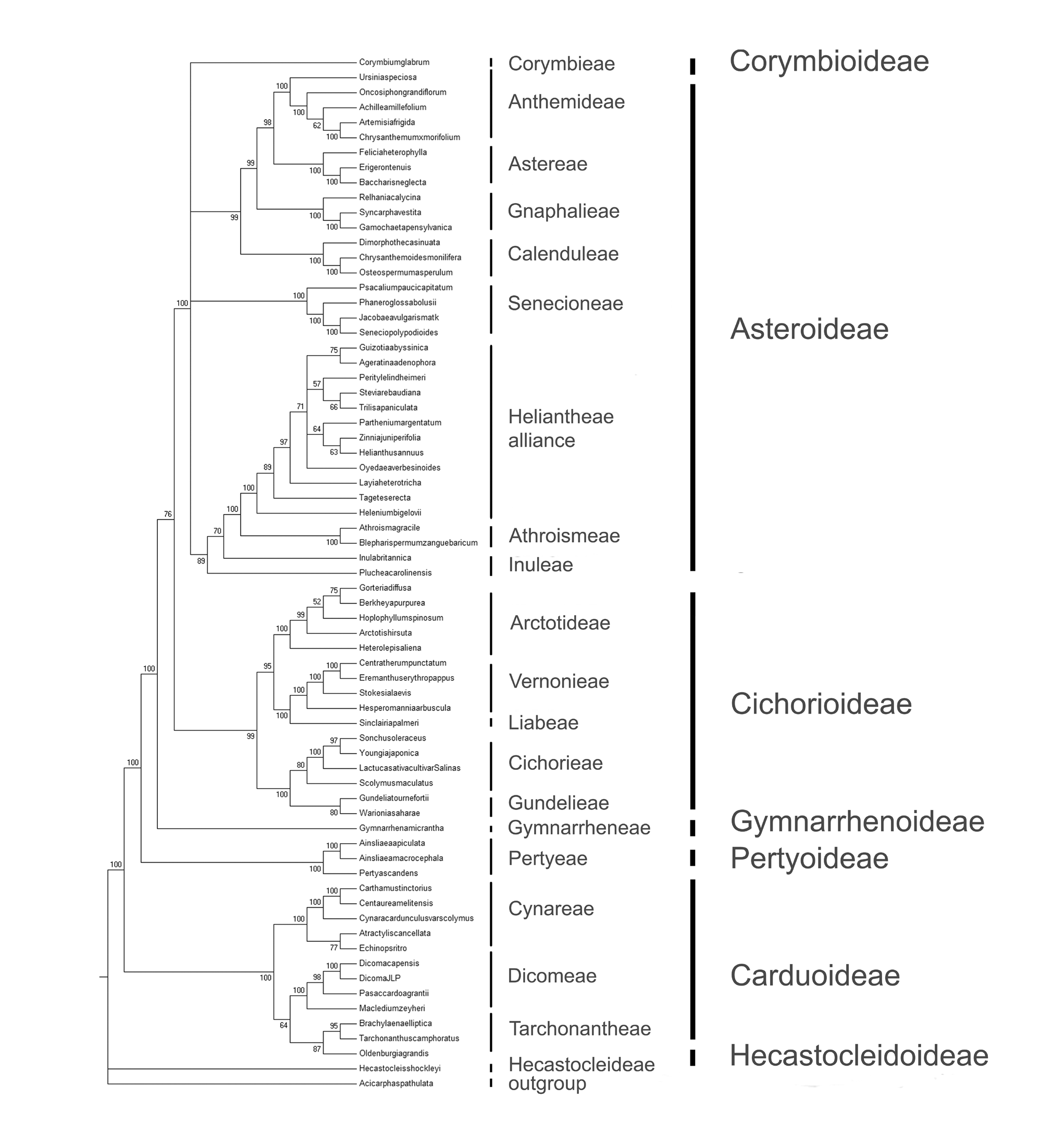

Supplement: S2 Fig — Seven coding regions were used: matk, ndhD, ndhF, ndhI, rbcL, rpoB and the first exon of rpoC1. The analysis was performed using RaxML Blackbox with the Gamma model of rate heterogeneity. Bootstrap support values were set greater than 50%. (TIF) [file pone.0120589.s002.tif]
