# Supplementary material for: Complete Chloroplast Genome of the Multifunctional Crop Globe Artichoke and Comparison with Other Asteraceae
Source: PLoS One. 2015 Mar 16;10(3):e0120589. doi: 10.1371/journal.pone.0120589 (PMC4361619; doi:10.1371/journal.pone.0120589)
Supplement: S1 Table — The name of primers refer to the cp regions amplified. (DOCX) [file pone.0120589.s003.docx]

**Table S1. Primer pairs used for BAC identification and junction validation**. The name of primers refer to the cp regions amplified

| **No.** | **Name** |  | **Sequence** | **Product length** |
| --- | --- | --- | --- | --- |
| 1 | Br1_BAC1 | F: | GGGCGTTAGAGCATTGAGAG | 299 bp |
|  | Br1_BAC1 | R: | ATGATAAACGGCTCGTCTCG |  |
| 2 | Br1_BAC2 | F: | CATTCTGAAACTAAGAAGAACTCC | 1220 bp |
|  | Br1_BAC2 | R: | TTTTCAGGAACAAGCAAATCA |  |
| 3 | LSC/IR_F | F: | TTACTCTTCGCGCTTTGTGA | 951 bp |
|  | LSC/IR_R | R: | GGGAAGGGGTTTTGATTGAT |  |
| 4 | IR/SSC_F | F: | TGCGTAATCTCAGCATTCAA | 1930 bp |
|  | IR/SSC_R | R: | TCCGGAGATTGCCCTAATAA |  |
| 5 | SSC/IR_F | F: | CAAGACGGGGTTAATTTGGA | 848 bp |
|  | SSC/IR_R | R: | GCCTCTGCATCTAGCACTGG |  |
| 6 | IR/LSC_F | F: | CCAAAAACTGCTCAGCAACA | 889 bp |
|  | IR/LSC_R | R: | GCTGCTATCGAAGCTCCATC |  |
