# Supplementary material for: Complete Chloroplast Genome of the Multifunctional Crop Globe Artichoke and Comparison with Other Asteraceae
Source: PLoS One. 2015 Mar 16;10(3):e0120589. doi: 10.1371/journal.pone.0120589 (PMC4361619; doi:10.1371/journal.pone.0120589)
Supplement: S2 Table — (DOCX) [file pone.0120589.s004.docx]

**Table S2** Codon-anticodon recognition pattern and codon usage for *Cynara cardunculus* var. *scolymus* chloroplast genome

| **AmAcid** | **Codon** | **Number** | **/1000** | **RSCU** | **tRNA** | **AmAcid** | **Codon** | **Number** | **/1000** | **RSCU** | **tRNA** |
| --- | --- | --- | --- | --- | --- | --- | --- | --- | --- | --- | --- |
| Ala | GCG | 153 | 5.82 | 0.43 | trnA-UGC | Pro | CCU | **413** | 15.70 | 1.50 | trnP-UGG |
| Ala | GCA | 411 | 15.63 | 1.15 |  | Pro | CCG | 165 | 6.27 | 0.60 |  |
| Ala | GCU | **635** | 24.14 | 1.78 |  | Pro | CCA | 328 | 12.47 | 1.19 |  |
| Ala | GCC | 223 | 8.63 | 0.64 |  | Pro | CCC | 195 | 7.42 | 0.71 |  |
| Cys | UGU | **211** | 8.02 | 1.44 | trnC-GCA | Gln | CAA | **711** | 27.03 | 1.53 | trnQ-UUG |
| Cys | UGC | 82 | 3.12 | 0.56 |  | Gln | CAG | 220 | 8.37 | 0.47 |  |
| Asp | GAU | **854** | 32.47 | 1.59 | trnD-GUC | Arg | CGU | 349 | 13.27 | 1.31 | trnR-ACG |
| Asp | GAC | 218 | 8.29 | 0.41 |  | Arg | CGG | 122 | 4.64 | 0.46 |  |
| Glu | GAA | **996** | 37.87 | 1.47 | trnE-UUC | Arg | CGA | 348 | 13.23 | 1.31 |  |
| Glu | GAG | 364 | 13.84 | 0.54 |  | Arg | CGC | 99 | 3.76 | 0.37 |  |
| Phe | UUU | **971** | 36.92 | 1.29 | trnF-GAA | Arg | AGA | **504** | 19.16 | 1.89 | trnR-UCU |
| Phe | UUC | 533 | 20.27 | 0.71 |  | Arg | AGG | 176 | 6.69 | 0.66 |  |
| Gly | GGU | 575 | 21.86 | 1.29 | trnG-UCC | Ser | UCU | **591** | 22.47 | 1.76 | trnS-GGA |
| Gly | GGG | 303 | 11.52 | 0.68 |  | Ser | UCA | 414 | 15.74 | 1.23 | trnS-UGA |
| Gly | GGA | **703** | 26.73 | 1.57 |  | Ser | UCG | 177 | 6.73 | 0.53 |  |
| Gly | GGC | 205 | 7.80 | 0.46 | trnG-GCC | Ser | AGU | 402 | 15.29 | 1.20 | trnS-GCU |
| His | CAU | **477** | 18.14 | 1.53 | trnH-GUG | Ser | AGC | 125 | 4.75 | 0.37 |  |
| His | CAC | 146 | 5.52 | 0.47 |  | Ser | UCC | 310 | 11.79 | 0.92 |  |
| Ile | AUA | 697 | 26.50 | 0.94 | trnI-CAU | Thr | ACA | 403 | 15.32 | 1.23 | trnT-UGU |
| Ile | AUU | **1080** | 41.06 | 1.46 | trnI-GAU | Thr | ACG | 133 | 5.06 | 0.41 |  |
| Ile | AUC | 446 | 16.96 | 0.60 |  | Thr | ACU | **525** | 19.96 | 1.61 | trnT-GGU |
| Lys | AAA | **1036** | 39.39 | 1.49 | trnK-UUU | Thr | ACC | 246 | 9.35 | 0.75 |  |
| Lys | AAG | 359 | 13.65 | 0.52 |  | Val | GUA | **542** | 20.61 | 1.52 | trnV-UAC |
| Leu | UUG | 583 | 22.17 | 1.25 | trnL-CAA | Val | GUG | 195 | 7.42 | 0.55 |  |
| Leu | UUA | **851** | 32.36 | 1.83 | trnL-UAA | Val | GUU | 510 | 19.39 | 1.43 | trnV-GAC |
| Leu | CUG | 182 | 6.92 | 0.39 | trnL-UAG | Val | GUC | 182 | 6.92 | 0.51 |  |
| Leu | CUA | 380 | 14.45 | 0.82 |  | Trp | UGG | **462** | 17.57 | 1 | trnW-CCA |
| Leu | CUU | 604 | 22.97 | 1.30 |  | Tyr | UAU | **805** | 30.61 | 1.61 | trnY-GUA |
| Leu | CUC | 192 | 7.30 | 0.41 |  | Tyr | UAC | 194 | 7.38 | 0.39 |  |
| Met | AUG | **619** | 23.54 | 1 | trnM-CAU | End | UGA | 17 | 0.65 | 0.59 |  |
|  |  |  |  |  | trnfM- CAU | End | UAG | 24 | 0.91 | 0.83 |  |
| Asn | AAU | **990** | 37.64 | 1.56 | trnN-GUU | End | UAA | **46** | 1.75 | 1.59 |  |
| Asn | AAC | 283 | 10.76 | 0.45 |  |  |  |  |  |  |  |

/1000: Relative frequency for a specific codon in 1000 codons.

RSCU: Relative Synonymous Codon Usage, measure of the extent of the non-random usage of synonymous codons for specific amino acids.

Bold character is referred to the most frequent codon
