# Supplementary material for: Complete Chloroplast Genome of the Multifunctional Crop Globe Artichoke and Comparison with Other Asteraceae
Source: PLoS One. 2015 Mar 16;10(3):e0120589. doi: 10.1371/journal.pone.0120589 (PMC4361619; doi:10.1371/journal.pone.0120589)
Supplement: S3 Table — (DOCX) [file pone.0120589.s005.docx]

**Table S3.** Direct and palindromic repeats in *C. cardunculus* var. *scolymus* cp genome

| No. | rep.type | length | copy no. | Position | Location | Genomic Region |
| --- | --- | --- | --- | --- | --- | --- |
| 1 | D | 11 | 2.7 | 11882/11893/11904 | *trnE(UUC)-rpoB* | spacer |
| 2 | D° | 12 | 2 | 22624/22636 | *rpoC2* | cds |
| 3 | D° | 12 | 2 | 24806/24818 | *atpI-atpH* | spacer |
| 4 | D° | 12 | 2 | 65196/65208 | *psbE-petL* | spacer |
| 5 | D° | 12 | 2 | 110920/110932 | *ycf1* | cds |
| 6 | D° | 16 | 2 | 54307/54323 | *atpB-rbcL* | spacer |
| 7 | D | 17 | 1.9 | 81119/81136 | *rpl16* | intron |
| 8 | D° | 24 | 2 | 76170/76194 | *petD* | intron |
| 9 | D | 18 | 2.1 | 46990/47008 | *trnL(UAA)-trnF(GAA)* | spacer |
| 10 | D | 18 | 3.5 | 90808/90826/90844 | *ycf2* | cds |
| 11 | D | 18 | 3.5 | 145238/145256/145274 | *ycf2* | cds |
| 12 | D° | 19 | 2 | 41612/41631 | *psbC-trnS(UGA)* | spacer |
| 13 | D° | 19 | 2 | 58415/58434 | *accD-psaI* | spacer |
| 14 | P° | 10 | 2 | 9198/9208 | *trnS(GCU)-trnC(GCA)* | spacer |
| 15 | P° | 10 | 2 | 20591/20601 | *rpoC2* | cds |
| 16 | D | 20 | 2.3 | 58613/58633 | *accD-psaI* | spacer |
| 17 | D° | 21 | 2 | 62596/62617 | *petA-psbJ* | spacer |
| 18 | D | 21 | 2 | 67470/67491 | *rps18* | cds |
| 19 | D | 21 | 2 | 112271/112292 | *ycf1* | cds |
| 20 | P | 11 | 2 | 6795/6806 | *rps16-trnQ(UUG)* | spacer |
| 21 | P | 11 | 2 | 56246/56257 | *rbcL-accD* | spacer |
| 22 | P | 11 | 2 | 116633/116644 | *ndhA* | intron |
| 23 | P° | 12 | 2 | 10347/10359 | *petN-psbM* | spacer |
| 24 | P | 12 | 2 | 48837/48849 | *ndhK* | cds |
| 25 | D° | 24 | 2 | 112485/112509 | *ycf1* | cds |
| 26 | P | 12 | 2 | 34416/34429 | *psbC-trnS(UGA)* | spacer |
| 27 | P | 12 | 2 | 63097/63110 | *petA-psbJ* | spacer |
| 28 | P° | 13 | 2 | 87235/87248 | *ycf2* | cds |
| 29 | P° | 13 | 2 | 148847/148860 | *ycf2* | cds |
| 30 | P | 14 | 2 | 30582/30597 | *trnR(UCU)-trnG(UCC)* | spacer |
| 31 | P | 14 | 2 | 46120/46134 | *trnT(UGU)-trnL(UAA)* | spacer |
| 32 | P* | 30 | 2 | 42821/75063 | *ycf3 2nd intron* | intron |
| 33 | D* | 30 | 2 | 75063/116973 | *petB* | intron |
| 34 | D | 30 | 3.5 | 112524/112554/112584 | *ycf1* | cds |
| 35 | D* | 30 | 2 | 124386/138134 | *rpl32-ndhF* | spacer |
| 36 | P* | 31 | 2 | 111049/113497 | *ycf1* | cds |
| 37 | D | 32 | 3 | 8444/34582/44539 | *psbI-trnS/trnS(GCU)* | spacer/gene |
| 38 | D | 32 | 1.9 | 106664/106696 | *rrn4.5-rrn5* | spacer |
| 39 | D | 32 | 1.9 | 129383/129415 | *rrn5-rrn4.5* | spacer |
| 40 | D* | 33 | 2 | 11729/30420 | *trnE(UUC)-rpoB* | spacer |
| 41 | D | 41 | 4 | 42818/97587/138480/116965 | *ycf3 2nd intron* | intron |
| 42 | D | 45 | 2 | 109447/109492 | *ycf1* | cds |
| 43 | P° | 24 | 2 | 73724/73748 | *psbT-psbN* | spacer |

Direct (D), palindromic (P) repeats in tandem or dispersed (*) distribution. Circles indicate perfect repeats, the others are imperfect. “Position” indicates the start nucleotide for each repeat. “Location” and “Genomic Region” refer to the first repeat.
