# Supplementary material for: Complete Chloroplast Genome of the Multifunctional Crop Globe Artichoke and Comparison with Other Asteraceae
Source: PLoS One. 2015 Mar 16;10(3):e0120589. doi: 10.1371/journal.pone.0120589 (PMC4361619; doi:10.1371/journal.pone.0120589)
Supplement: S4 Table — (DOCX) [file pone.0120589.s006.docx]

**Table S4** SSRs found in artichoke cp genome

| No. | SSR | Repeats | Length | Start | End | Location | Genome region |
| --- | --- | --- | --- | --- | --- | --- | --- |
| 1 | A | 8 | 8 | 131 | 138 | *trnH(GUG)-psbA* | spacer |
| 2 | T | 8 | 8 | 1976 | 1983 | *trnK(UUU) intron* | intron |
| 3 | T | 9 | 9 | 2305 | 2313 | *trnK(UUU) intron* | intron |
| 4 | T | 9 | 9 | 3795 | 3803 | *matK-trnK(UUU)* | spacer |
| 5 | A | 13 | 13 | 4418 | 4430 | *matK-trnK(UUU)* | spacer |
| 6 | A | 8 | 8 | 4760 | 4767 | *matK-trnK(UUU)* | spacer |
| 7 | C | 8 | 8 | 5391 | 5398 | *rps16 intron* | intron |
| 8 | A | 8 | 8 | 6444 | 6451 | *rps16-trnQ(UUG)* | spacer |
| 9 | A | 9 | 9 | 9498 | 9506 | *trnC(GCA)-petN* | spacer |
| 10 | T | 8 | 8 | 10624 | 10631 | *psbM-trnD(GUC)* | spacer |
| 11 | A | 8 | 8 | 12198 | 12205 | *trnE(UUC)-rpoB* | spacer |
| 12 | A | 10 | 10 | 13123 | 13132 | *rpoB* | cds |
| 13 | A | 8 | 8 | 13229 | 13236 | *rpoB* | cds |
| 14 | A | 8 | 8 | 16354 | 16361 | *rpoC1 intron* | intron |
| 15 | T | 8 | 8 | 16981 | 16988 | *rpoC1 2nd exon* | cds |
| 16 | A | 9 | 9 | 17745 | 17753 | *rpoC1 2nd exon* | cds |
| 17 | A | 10 | 10 | 18170 | 18179 | *rpoC1 2nd exon* | cds |
| 18 | AT | 5 | 10 | 18394 | 18403 | *rpoC1 2nd exon* | cds |
| 19 | AT | 5 | 10 | 19390 | 19399 | *rpoC2* | cds |
| 20 | T | 9 | 9 | 20628 | 20636 | *rpoC2* | cds |
| 21 | A | 8 | 8 | 22665 | 22672 | *rpoC2* | cds |
| 22 | T | 9 | 9 | 22978 | 22986 | *rpoC2-rps2* | spacer |
| 23 | T | 8 | 8 | 24702 | 24709 | *atpI* | cds |
| 24 | A | 8 | 8 | 24740 | 24747 | *atpI-atpH* | spacer |
| 25 | A | 9 | 9 | 24853 | 24861 | *atpI-atpH* | spacer |
| 26 | T | 10 | 10 | 25793 | 25802 | *atpI-atpH* | spacer |
| 27 | AT | 7 | 14 | 26351 | 26364 | *atpH-atpF* | spacer |
| 28 | T | 8 | 8 | 29603 | 29610 | *trnR(UCU)-trnG(UCC)* | spacer |
| 29 | A | 8 | 8 | 29895 | 29902 | *trnG(UCC)intron* | intron |
| 30 | T | 8 | 8 | 30734 | 30741 | *trnT(GGU)-psbD* | spacer |
| 31 | TAA | 4 | 12 | 30778 | 30789 | *trnT(GGU)-psbD* | spacer |
| 32 | A | 8 | 8 | 31822 | 31829 | *trnT(GGU)-psbD* | spacer |
| 33 | TTC | 4 | 12 | 34229 | 34240 | *psbC* | cds |
| 34 | A | 10 | 10 | 34416 | 34425 | *psbC-trnS(UGA)* | spacer |
| 35 | T | 12 | 12 | 34431 | 34442 | *psbC-trnS(UGA)* | spacer |
| 36 | A | 8 | 8 | 35298 | 35305 | *psbZ-trnG(UCC)* | spacer |
| 37 | A | 8 | 8 | 35346 | 35353 | *psbZ-trnG(UCC)* | spacer |
| 38 | T | 8 | 8 | 35739 | 35746 | *trnG(UCC)-trnfM(CAU)* | spacer |
| 39 | T | 8 | 8 | 36276 | 36283 | *rps14* | cds |
| 40 | C | 8 | 8 | 39165 | 39172 | *psaA* | cds |
| 41 | T | 10 | 10 | 41366 | 41375 | *psaA-ycf3* | spacer |
| 42 | T | 8 | 8 | 43398 | 43405 | *ycf3 1st intron* | intron |
| 43 | A | 9 | 9 | 43463 | 43471 | *ycf3 1st intron* | intron |
| 44 | A | 10 | 10 | 43711 | 43720 | *ycf3-trnS(GGA)* | spacer |
| No. | SSR | Repeats | Length | Start | End | Location | Genome region |
| 45 | T | 9 | 9 | 45755 | 45763 | *rps4-trnT(UGU)* | spacer |
| 46 | T | 9 | 9 | 46092 | 46100 | *trnT(UGU)-trnL(UAA)* | spacer |
| 47 | T | 14 | 14 | 46118 | 46131 | *trnT(UGU)-trnL(UAA)* | spacer |
| 48 | T | 8 | 8 | 47952 | 47959 | *trnF(GAA)-ndhJ* | spacer |
| 49 | T | 13 | 13 | 49936 | 49948 | *ndhC-trnV(UAC)* | spacer |
| 50 | T | 9 | 9 | 54247 | 54255 | *atpB-rbcL* | spacer |
| 51 | A | 8 | 8 | 54389 | 54396 | *atpB-rbcL* | spacer |
| 52 | T | 8 | 8 | 56777 | 56784 | *accD* | cds |
| 53 | A | 9 | 9 | 58277 | 58285 | *accD-psaI* | spacer |
| 54 | AAT | 4 | 12 | 58508 | 58519 | *accD-psaI* | spacer |
| 55 | T | 10 | 10 | 58860 | 58869 | *psaI-ycf4* | spacer |
| 56 | T | 8 | 8 | 59412 | 59419 | *ycf4* | cds |
| 57 | TTC | 4 | 12 | 62433 | 62444 | *petA* | cds |
| 58 | T | 13 | 13 | 62761 | 62773 | *petA-psbJ* | spacer |
| 59 | A | 8 | 8 | 63216 | 63223 | *petA-psbJ* | spacer |
| 60 | T | 11 | 11 | 64592 | 64602 | *psbE-petL* | spacer |
| 61 | A | 16 | 16 | 65009 | 65024 | *psbE-petL* | spacer |
| 62 | TA | 6 | 12 | 67339 | 67350 | *rpl33-rps18* | spacer |
| 63 | TATT | 3 | 12 | 67395 | 67406 | *rpl33-rps18* | spacer |
| 64 | A | 8 | 8 | 67877 | 67884 | *rps19-rpl20* | spacer |
| 65 | A | 11 | 11 | 68620 | 68630 | *rpl20-rps12* | spacer |
| 66 | A | 9 | 9 | 70622 | 70630 | *clpP 1st intron* | intron |
| 67 | T | 10 | 10 | 70974 | 70983 | *clpP 1st intron* | intron |
| 68 | T | 8 | 8 | 71134 | 71141 | *clpP 1st intron* | intron |
| 69 | T | 9 | 9 | 73498 | 73506 | *psbB-psbT* | spacer |
| 70 | A | 10 | 10 | 77329 | 77338 | *petD-rpoA* | spacer |
| 71 | T | 10 | 10 | 77575 | 77584 | *rpoA* | cds |
| 72 | T | 11 | 11 | 80007 | 80017 | *rps8-rpl14* | spacer |
| 73 | T | 9 | 9 | 80499 | 80507 | *rpl14-rpl16* | spacer |
| 74 | T | 10 | 10 | 80584 | 80593 | *rpl14-rpl16* | spacer |
| 75 | TTCT | 3 | 12 | 81764 | 81775 | *rpl16 intron* | intron |
| 76 | T | 10 | 10 | 82087 | 82096 | *rpl16-rps3* | spacer |
| 77 | T | 9 | 9 | 82882 | 82890 | *rpl22* | cds |
| 78 | T | 8 | 8 | 83584 | 83591 | *rps19* | cds |
| 79 | T | 9 | 9 | 83616 | 83624 | *rps19* | cds |
| 80 | T | 8 | 8 | 83651 | 83658 | *rps19-rpl2* | spacer |
| 81 | A | 8 | 8 | 86266 | 86273 | *ycf2* | cds |
| 82 | A | 8 | 8 | 87889 | 87896 | *ycf2* | cds |
| 83 | T | 9 | 9 | 88221 | 88229 | *ycf2* | cds |
| 84 | A | 9 | 9 | 89033 | 89041 | *ycf2* | cds |
| 85 | T | 9 | 9 | 101952 | 101960 | *trnI(GAU) intron* | intron |
| 86 | A | 11 | 11 | 107024 | 107034 | *rrn5-trnR(ACG)* | spacer |
| 87 | GAA | 5 | 15 | 108956 | 108970 | *ycf1* | cds |
| 88 | GTTT | 3 | 12 | 109067 | 109078 | *ycf1* | cds |
| 89 | T | 8 | 8 | 109097 | 109104 | *ycf1* | cds |
| 90 | T | 8 | 8 | 109189 | 109196 | *ycf1* | cds |
| No. | SSR | Repeats | Length | Start | End | Location | Genome region |
| 91 | A | 8 | 8 | 109719 | 109726 | *ycf1* | cds |
| 92 | A | 8 | 8 | 109797 | 109804 | *ycf1* | cds |
| 93 | A | 9 | 9 | 110296 | 110304 | *ycf1* | cds |
| 94 | A | 9 | 9 | 111122 | 111130 | *ycf1* | cds |
| 95 | A | 9 | 9 | 111145 | 111153 | *ycf1* | cds |
| 96 | ATA | 4 | 12 | 111159 | 111170 | *ycf1* | cds |
| 97 | A | 8 | 8 | 111322 | 111329 | *ycf1* | cds |
| 98 | T | 8 | 8 | 111471 | 111478 | *ycf1* | cds |
| 99 | AATC | 3 | 12 | 111769 | 111780 | *ycf1* | cds |
| 100 | A | 9 | 9 | 111848 | 111856 | *ycf1* | cds |
| 101 | A | 8 | 8 | 112145 | 112152 | *ycf1* | cds |
| 102 | A | 9 | 9 | 113323 | 113331 | *ycf1* | cds |
| 103 | A | 9 | 9 | 114088 | 114096 | *rps15* | cds |
| 104 | GATT | 3 | 12 | 116222 | 116233 | *ndhA intron* | intron |
| 105 | A | 8 | 8 | 116579 | 116586 | *ndhA intron* | intron |
| 106 | TTTC | 3 | 12 | 116628 | 116639 | *ndhA intron* | intron |
| 107 | AG | 5 | 10 | 116647 | 116656 | *ndhA intron* | intron |
| 108 | T | 8 | 8 | 120071 | 120078 | *psaC-ndhD* | spacer |
| 109 | A | 8 | 8 | 121058 | 121065 | *ndhD* | cds |
| 110 | A | 9 | 9 | 121709 | 121717 | *ndhD-ccsA* | spacer |
| 111 | A | 8 | 8 | 122257 | 122264 | *ccsA* | cds |
| 112 | A | 8 | 8 | 123155 | 123162 | *trnL(UAG)-rpl32* | spacer |
| 113 | A | 10 | 10 | 123293 | 123302 | *trnL(UAG)-rpl32* | spacer |
| 114 | T | 9 | 9 | 123750 | 123758 | *trnL(UAG)-rpl32* | spacer |
| 115 | ATAA | 3 | 12 | 124405 | 124416 | *rpl32-ndhF* | spacer |
| 116 | A | 8 | 8 | 126266 | 126273 | *ndhF* | cds |
| 117 | T | 8 | 8 | 126659 | 126666 | *ndhF* | cds |
| 118 | T | 8 | 8 | 127005 | 127012 | *ndhF* | cds |
| 119 | T | 11 | 11 | 129074 | 129084 | *trnR(ACG)-rrn5* | spacer |
| 120 | A | 9 | 9 | 134148 | 134156 | *trnI(GAU)intron* | intron |
| 121 | T | 9 | 9 | 147067 | 147075 | *ycf2* | cds |
| 122 | A | 9 | 9 | 147879 | 147887 | *ycf2* | cds |
| 123 | T | 8 | 8 | 148212 | 148219 | *ycf2* | cds |
| 124 | T | 8 | 8 | 149835 | 149842 | *ycf2* | cds |
| 125 | A | 8 | 8 | 152450 | 152457 | *rpl2-rps19* | spacer |
| 126 | A | 9 | 9 | 152484 | 152492 | *rps19* | pseudo |
| 127 | A | 8 | 8 | 152517 | 152524 | *rps19* | pseudo |
