# Supplementary material for: Complete Chloroplast Genome of the Multifunctional Crop Globe Artichoke and Comparison with Other Asteraceae
Source: PLoS One. 2015 Mar 16;10(3):e0120589. doi: 10.1371/journal.pone.0120589 (PMC4361619; doi:10.1371/journal.pone.0120589)
Supplement: S5 Table — Sequences were designed on the most parsimony-informative coding regions revealed by the multialignment of the nine Asteraceae complete cp genomes. (DOCX) [file pone.0120589.s007.docx]

**Table S5. Barcoding primer pair candidates**. Sequences were designed on the most parsimony-informative coding regions revealed by the multialignment of the nine Asteraceae complete cp genomes.

| **No.** | **Region** |  | **Sequence** | **Product length** | **Pars. inf.** | **Pars. uninf.** |
| --- | --- | --- | --- | --- | --- | --- |
| 1 | *ccsA* | F: | TTKACCCCAAAATAGCATATCCA | 725 | 37 | 53 |
|  |  | R: | GCCATTTBCCRCTAAGTGATTTAT |  |  |  |
| 2 | *matK* | F: | CGTACAGTACTTTTATGCTTACGAGC | 876 | 40 | 76 |
|  |  | R: | CTTGGTTCAGGCTCTTCGCTAT |  |  |  |
| 3 | *accD* | F: | AAGATGGTGGTTMAATTCGATGT | 840 | 44 | 98 |
|  |  | R: | GGATCCARAGAGACCATGTCTTC |  |  |  |
| 4 | *ndhA* | F: | GGCCTTCTTATGTCAGGATATGG | 608 | 31 | 38 |
|  |  | R: | GAATCKTGGGARGTACTCCTTGA |  |  |  |
| 5 | *ycf1* | F: | GAATCCATGGAAAAMCTGGTTAAGA | 751 | 79 | 93 |
|  |  | R: | GCTTGGRATAAACCAAGGKTTCT |  |  |  |
| 6 | *clpP* | F: | KGGTMTCATCCTCRATACTGAGAT | 954 | 44 | 91 |
|  |  | R: | CGAARTSCTGGAGAGGAAGAT |  |  |  |
| 7 | *rbcL* | F: | ATGTCACCACAAACAGAGACTAAAGC | 712 | 26 | 20 |
|  |  | R: | GCCCTTTGATTTCACCTGTTTC |  |  |  |
| 8 | *rps16* | F: | GGAAATAGAGAATMGCRGGAACG | 995 | 72 | 105 |
|  |  | R: | TAAAACGATGTGGTAGAAAGCAAC |  |  |  |

Pars.: parsimony

Uninf.: uninformative

Inf.: informative
